# SARS‐CoV‐2 nucleocapsid protein variants have differential RNA chaperone activity

**DOI:** 10.1111/febs.70329

**Published:** 2025-11-20

**Authors:** Sabrina Babl, Julia M. Seidel, Fabian Kugler, Elisabeth Silberhorn, Anna Ludwig, Jonas Abel, Sophia Winklbauer, Niklas Schaub, Silvia Materna‐Reichelt, Kamran Honarnejad, Nataša Stojanović Gužvić, Gernot Längst

**Affiliations:** ^1^ Biochemistry Center Regensburg University of Regensburg Germany; ^2^ Fraunhofer Institute for Toxicology and Experimental Medicine ITEM‐R Personalized Tumor Therapy Regensburg Germany

**Keywords:** nucleocapsid, Omicron, RNA‐chaperone, SARS‐CoV‐2

## Abstract

The single‐stranded RNA genome of the SARS‐CoV‐2 virus is characterized by a complex secondary structure formed by patches of intramolecular RNA double‐strands. Here, we show that the nucleocapsid (N) protein is not only the specific viral RNA packaging protein, but also acts as an RNA chaperone, facilitating RNA folding. RNA chaperones are classified by their non‐specific RNA binding and the presence of intrinsically disordered regions (IDRs). N possesses three IDRs, separated by the structured RNA‐binding domain (RBD) and the C‐terminal domain (CTD). Our study identifies the amino acids 46–364 (RBD–IDR2–CTD) as crucial for chaperone activity, with flanking IDRs either enhancing or repressing this function, revealing the essential role of IDRs for the chaperone mechanism. Furthermore, a comparison between the Wuhan and Omicron BA.5 variant N shows reduced chaperone activity of the Omicron N protein. However, mimicking the cellular phosphorylation state of Omicron N restored its chaperone activity to the levels of the Wuhan variant. Our results identify N‐phosphorylation as a regulatory mechanism of chaperone activity, emphasizing an intricate regulatory role of post‐translational modifications in the dynamics of viral RNA secondary structure establishment. The regulation of RNA chaperoning could serve as a potential therapeutic target for future treatment of RNA viruses.

## Graphical abstract

The SARS‐CoV‐2 nucleocapsid (N) protein is not only the specific viral RNA packaging protein, but also acts as an RNA chaperone, facilitating RNA folding. We show that it facilitates RNA annealing through its RBD–IDR2–CTD region, identifying it as a functional RNA chaperone. The Omicron BA.5 variant of the N protein shows reduced chaperone activity compared to the Wuhan wild‐type variant. Although phosphorylation has no effect on wild‐type N, phosphomimetic modification of BA.5 N restores its RNA chaperone function to wild‐type levels, indicating a regulatory role of phosphorylation in variant‐specific RNA‐folding efficiency.

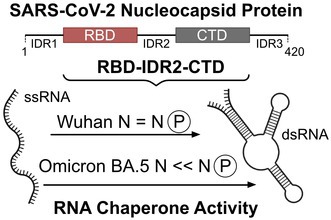

**Abbreviations**
BH: Benjamini–Hochberg
CTD: C‐terminal domain
E: envelope
EMSA: electromobility shift assay
GSK‐3: glycogen synthetase kinase‐3
HIV‐1: human immunodeficiency virus 1
IDR: intrinsically disordered region
kb: kilobases
K_D_
: dissociation constant
LLPS: liquid–liquid phase separation
M: membrane
MST: microscale thermophoresis
N: nucleocapsid
nanoDSF: nano differential scanning fluorimetry
Ncp7: nucleocapsid protein 7
PAA: polyacrylamide
PM: phosphomimetic
RBD: RNA‐binding domain
S: spike
SARS‐CoV‐2: severe acute respiratory syndrome coronavirus 2
SD: standard deviation
StpA: suppressor of td phenotype A
T_M_
: melting temperature
WT: wild‐type
Zn: zinc

## Introduction

RNA molecules are central players in various cellular processes, carrying out essential functions such as being the templates and major regulators of translation, and actively regulating gene expression [1]. Additionally, RNAs function as catalysts in the form of ribozymes, playing essential roles in nuclear architecture and chromatin organization [2]. For many viruses, RNA serves as the hereditary molecule, either in the form of a double‐stranded (ds) or highly structured single‐stranded (ss) RNA genome [3].

The presence of RNA genomes in viruses not only provides flexibility in terms of genome size and structure but also allows viruses to acquire genetic diversity, allowing fast adaptations to new hosts, evading host immune responses, and developing resistance to antiviral drugs, as these exhibit a strongly increased mutation rate compared to DNA genomes [4]. The higher mutation rate in RNA genomes is primarily due to the error‐prone nature of RNA‐dependent RNA polymerases and their lack of proofreading mechanisms, as well as the single‐stranded nature of RNA, which contributes mainly to its flexibility and adaptation capabilities [5, 6].

Single‐stranded RNA molecules can adopt various structural conformations by exploiting different intramolecular base pair interactions, resulting in specific RNA folds. Additionally, the presence of non‐canonical base pairs, tertiary interactions, and long‐range interactions further expands the potential structural outcomes. RNA folding is a complex process influenced by various factors, including the sequence of nucleotides, thermodynamic stability, and kinetic pathways, leading to many different, stable RNA structures. The folding process is not solely determined by thermodynamics, but kinetic factors and associated proteins also play a role. RNA molecules can get trapped in kinetic intermediates, forming different metastable structures and off‐pathway folding traps [7, 8, 9]. However, as the correct RNA structure is essential for its proper biological function and interaction with other molecules, proteins evolved to resolve the folding traps and promote proper RNA folding [10, 11]. These are either specific RNA‐binding proteins, which stabilize the RNA structure by forming a stable RNA–protein complex, RNA helicases, which facilitate the unwinding of RNA in an ATP‐dependent mechanism or so‐called RNA chaperones, which dynamically change the RNA structure like helicases [7, 8]. RNA chaperones assist the proper folding of RNA molecules, exhibiting a crucial role in structural biogenesis and function [12, 13]. These proteins commonly lack sequence/structure specificity, act through transient RNA interactions, and do not require energy for their function [14]. Unlike many protein chaperones, which require ATP as an energy source, RNA chaperones go through cycles of transient unfolding and folding without requiring ATP [15]. An intriguing feature shared by RNA chaperones is the presence of intrinsically disordered regions (IDRs) [16, 17]. IDRs are functional protein domains lacking a stable three‐dimensional structure, often involved in protein–protein and protein–RNA interactions. The IDRs of RNA chaperones account for more than half of their amino acid residues, an unprecedented occurrence compared to other protein classes, underscoring their crucial role in RNA chaperoning [17]. The IDRs likely exhibit different functions in chaperones, either as molecular recognition elements by acting as solubilizers and preventing aggregation of the misfolded substrate (typically for protein chaperones), or locally loosening the structure of the kinetically trapped RNA folding intermediate to facilitate its folding into the correct conformational state [16]. It is suggested that a two‐step entropy transfer mechanism drives RNA folding toward a thermodynamically stable and functional structure. In the first step, entropy is transferred from the protein to the substrate: the RNA chaperone binds to the misfolded RNA, re‐organizing the IDRs of the protein, and unfolding the RNA. In the second step, the entropy transfer occurs in the opposite direction, from the substrate to the protein: the RNA folds and the chaperone's IDRs become unfolded again. These steps occur in many cycles of order–disorder until the proper folded RNA substrate is released [16, 18]. IDRs were also shown to be drivers of liquid–liquid phase separation (LLPS) and required to form RNA granules by interacting cooperatively with the folded domains of the protein [19].

The number of identified RNA chaperones is steadily growing, and they can be found in all kingdoms of life, ranging from prokaryotic to higher eukaryotic organisms, as well as viruses, indicating their fundamental role in RNA metabolism and processing [20]. Within viruses, RNA chaperones have been identified in several viral families, including retroviruses, enteroviruses, and coronaviruses [14, 21]. The first RNA chaperone discovered was the nucleocapsid protein 7 (Ncp7) of the human immunodeficiency virus type 1 (HIV‐1) [22, 23]. RNA dimerization, encapsidation, and the annealing of the primer tRNA to the initiation site of reverse transcription are promoted by Ncp7 [24]. Ncp7 contains two Zinc (Zn) finger motifs, which are linked by basic amino acids and flanked by a highly basic N terminus and a short C terminus. An *in vivo* study investigating Ncp7 mutations showed both Zn finger motifs as well as the N terminus and the linker being involved in RNA dimerization of the HIV‐1 genome by a so‐called RNA–RNA kissing mechanism [25].

The nucleocapsid (N) protein of SARS‐CoV‐2 has been described promoting viral RNA sequences to form hybrids by annealing nucleic acids and destabilizing RNA duplexes [26, 27]. The N protein of SARS‐CoV‐2 was also suggested to be a viral RNA chaperone [28]. However, functional proof and mechanistic studies are still missing.

SARS‐CoV‐2 was first detected at the end of 2019 in Wuhan, China. Since then, this newly emerged coronavirus has rapidly spread worldwide, causing the global COVID‐19 pandemic. SARS‐CoV‐2 has not disappeared since then, but currently, among others, the newly evolved Omicron JN.1 strain is spreading at a high rate, showing the continuous health risk of this virus [29]. SARS‐CoV‐2 is a positive‐sense ssRNA virus that belongs to the family of beta‐coronaviruses with a genome size of approximately 30 kilobases (kb). On its 5′ end, non‐structural and accessory proteins are encoded, whereas the 3′ end of the genome harbors the coding sequences for the four structural proteins. Among those four structural proteins, the spike (S), membrane (M), and envelope (E) proteins are associated with the viral surface, whereas the N protein is located inside the virus, packaging the viral genome [30, 31].

The N protein has many additional functions in the virus besides RNA packaging. For example, N acts as a general regulator of viral gene replication and expression and inhibits the host's RNA interference defense mechanism. Furthermore, N protein regulates host–cell interactions and induces immune responses [31]. The SARS‐CoV‐2 N protein plays a critical role in the viral life cycle, being the most highly expressed protein in infected cells, binding to the viral RNA genome, and forming a ribonucleoprotein complex that can be packaged into the viral particle. This interaction between the N protein and the viral RNA is essential for the assembly and stability of the viral nucleocapsid [31, 32]. Structural characterization of the SARS‐CoV‐2 N protein reveals a heterogeneous, 419‐amino acid long, multi‐domain RNA‐binding protein, existing as a homodimer. It contains two well‐conserved folded domains, namely the N‐terminal domain (NTD), being the predicted RNA‐binding domain (RBD) of the protein, and the C‐terminal domain (CTD), which serves as the oligomerization domain. These structured domains are flanked and separated by IDRs, with the central IDR harboring a serine/arginine‐rich region that can be phosphorylated [31, 33, 34]. During the viral life cycle, the N protein is phosphorylated by cellular kinases, like glycogen synthase kinase‐3 (GSK‐3), exhibiting regulatory functions in viral replication, transcription, and genome packaging. Furthermore, N phosphorylation is being proposed as a cellular response mechanism to SARS‐CoV‐2 infection [35, 36].

In this study, we identified and characterized the RNA chaperone activity of the SARS‐CoV‐2 N protein, revealing the specific protein domains driving this mechanism. Moreover, we show the role of N phosphorylation on its RNA hybridization activity, shedding light on its regulatory role in viral replication and transcription. Our data reveal the proficient nucleic acid annealing activity of the SARS‐CoV‐2 N protein by an RNA chaperoning mechanism, demonstrating the central contribution of IDR2 to this process, as it synergistically cooperates in RNA annealing with the RBD and CTD domains. Notably, the N protein of the BA.5 Omicron lineage of CoV‐2 exhibits a strongly reduced RNA chaperoning activity compared to the Wuhan strain protein. However, phosphorylation of the BA.5 N protein fully restores its chaperone activity, while no change in activity was observed in the Wuhan strain N protein. Understanding the RNA chaperoning function of the SARS‐CoV‐2 N protein and the impact of phosphorylation provides mechanistic insights into viral replication and RNA‐related pathogenesis. Furthermore, uncovering the complexity of RNA–protein interactions and the analysis of the RNA chaperoning mechanism could open new avenues for targeted antiviral drug development.

## Results

### Double‐stranded RNA is the preferred substrate for the SARS‐CoV‐2 nucleocapsid protein

The nucleocapsid (N) protein of SARS‐CoV‐2 plays a crucial role in viral RNA packaging, thereby facilitating the assembly of new virions during infection. While the N protein's ability to bind to single‐stranded RNA (ssRNA) and its involvement in LLPS have been well documented [37], its potential role in genome folding and binding as an RNA chaperone has not been fully elucidated.

**Fig. 1:**
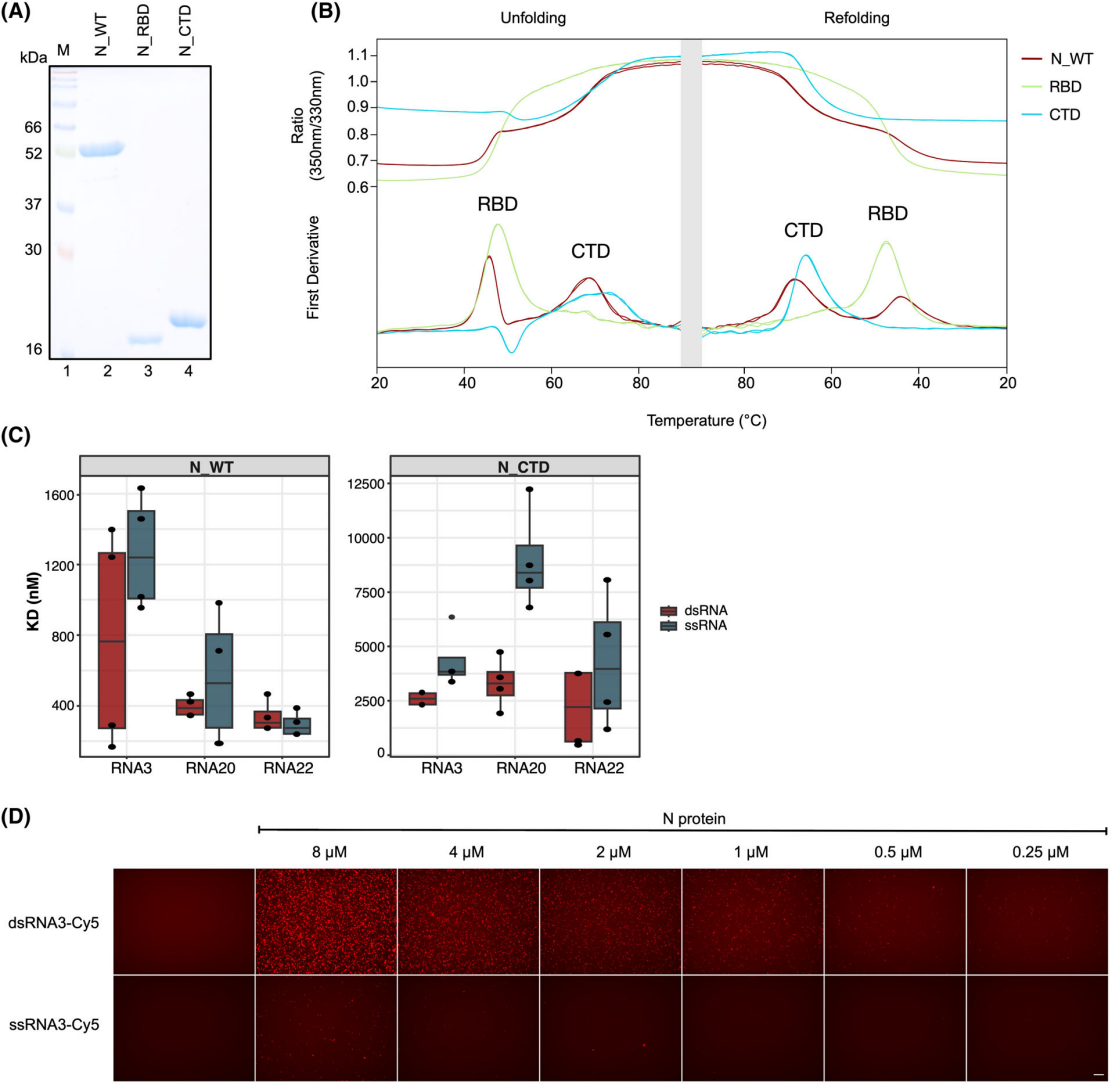
N protein stability and binding preferences. (A) Recombinant full‐length wild‐type N protein (N_WT), the receptor‐binding domain (N_RBD), and the C‐terminal domain (N_CTD) were expressed in *E. coli* and purified using a 6×His tag. Equal amounts (2 μg) of each protein were resolved on a 15% SDS gel. (B) The stability and quality of the purified proteins were assessed by differential scanning fluorometry (nanoDSF). The intrinsic tryptophan fluorescence of 10 μm of each protein was measured at 330 nm and 350 nm over a temperature gradient from 20 °C to 95 °C and vice versa (x‐axis). The y‐axis represents the rate of fluorescence ratio change per degree Celsius (°C). The RBD unfolding peak was observed at 45 °C, while the CTD unfolded at 68 °C. Moreover, all proteins showed proper refolding ability after heat denaturation. Number of replicates *n* = 4. (C) Comparative boxplots showing the binding affinities of three distinct SARS‐CoV‐2 RNAs (RNA3, RNA20, RNA22) in their single‐stranded (blue‐gray) and double‐stranded (red) forms with N_WT and N_CTD. Data are derived from MST analysis. Each boxplot summarizes four MST measurements, illustrating the interquartile range (IQR) with the median denoted by a horizontal line within the box. The whiskers extend to a maximum of 1.5 times the IQR above and below the upper and lower quartiles, respectively. Data points beyond this range are marked as outliers. The x‐axis categorizes the different RNAs, while the y‐axis represents the *K*_D_ values, with distinct scaling between N_WT and N_CTD. Notably, for each RNA, dsRNA exhibits comparable or higher binding affinity than ssRNA. Number of replicates *n* = 4. (D) Droplet formation assays were performed by mixing various concentrations of full‐length wild‐type N protein with either ssRNA3 or dsRNA3 (labeled with Cy5). After incubation at 25 °C for 2 h, droplet formation was visualized by fluorescent microscopy (40× magnification). For ssRNA, phase separation and droplet formation were detected starting at 2 μm of N, for dsRNA at 250 nM of N protein, respectively. Scale bar = 25 μm. Number of replicates *n* = 4.

To explore this, we expressed and purified recombinant full‐length Wuhan N protein (N_WT), as well as its individual structured domains, the RNA‐binding domain (RBD), and the C‐terminal domain (CTD) (Fig. 1A). Protein folding and stability were confirmed by nano differential scanning fluorimetry (nanoDSF) analysis, which monitors protein unfolding by detecting the intrinsic tryptophan or tyrosine fluorescence at 330 and 350 nm over a temperature gradient. In our studies on the SARS‐CoV‐2 N protein, we observe two distinct unfolding transitions in the first derivative of the fluorescence signal. The first peak revealed a thermal melting temperature (*T*_M_) of 45 °C for the RBD and 68 °C for the CTD, and their capability to refold (Fig. 1B). These data reveal properly folded protein batches of high purity.

To assess the RNA‐binding affinity, we used microscale thermophoresis (MST) to quantify the interactions between the protein deletion constructs and, three different 26‐nt long RNAs derived from distinct locations within the viral genome (RNA3, RNA20, RNA22). Binding affinities were determined for the single‐stranded and double‐stranded forms of these RNAs. The measured dissociation constants (*K*_D_) are summarized as a boxplot (Fig. 1C), with corresponding MST curves and raw data shown in the Fig. S1A,C,D. In addition, validation of RNA binding was performed by complementary electromobility shift assays (EMSAs) (Fig. S1B).

The experiments unveiled that N_WT binds ssRNA with a *K*_D_ of 1259 nM and dsRNA with a *K*_D of_ 647 nM, indicating a higher binding affinity for dsRNA. However, it is noteworthy that binding properties are not identical for all RNA sequences and the standard deviation (SD) for dsRNA3 measurements was relatively high, likely due to the cooperative binding behavior of N_WT, as shown by EMSA. This behavior would typically be better characterized using Hill‐based EC_50_ models. However, to allow direct comparison with N_CTD and N_RBD, which do not exhibit cooperative binding under these conditions, we opted to report *K*_D_ values. Importantly, EC_50_‐based analysis yielded similar binding affinities (data not shown). N_CTD exhibits a significant RNA‐binding activity, albeit with lower binding affinities than the full‐length N protein (*K*_D_'s of 4288 nM for ssRNA3 and 2554 nM for dsRNA3). A comparable trend is observed for RNA20, with N_WT exhibiting a *K*_D_ of 425 nM to ssRNA20 and 398 nM to dsRNA20, whereas N_CTD displayed a *K*_D_ of 8535 nM to ssRNA20 and 3300 nM to dsRNA20. Interestingly, for RNA22, the binding affinity N_WT does not change when using dsRNA. In contrast, we detected enhanced binding affinity of N_CTD to dsRNA20 and the other double‐stranded substrates. Using the RNAs of 26 nt length and our reaction conditions, revealed only very low RNA‐binding affinities of the RBD. The MST assay indicated RNA binding, but measurements did not reach a plateau at the protein concentrations used. Accordingly, we can only state that RNA binding is indicated, with binding affinities above 20.000 nM (Fig. S1A,C).

These results support previous findings that multiple domains contribute to RNA binding in coronavirus N proteins [38]. Notably, our data reveal a novel role for the CTD as a functional RNA‐binding domain, with a two‐ to threefold higher affinity for dsRNA than ssRNA, clearly surpassing the RNA‐binding capability of the RBD. Our results show that RNA‐binding domains cooperate to achieve cooperative, high‐affinity binding in the full‐length N protein. To further address the preferential dsRNA binding in a complementary assay, we performed LLPS assays with the same, short ssRNA and dsRNA molecules. These assays confirmed the preferential dsRNA binding by N, requiring an order of magnitude lower concentrations of dsRNA for LLPS formation (Fig. 1D). While LLPS with ssRNA occurred at approximately 4 μm of N protein, dsRNA droplets were detectable at 250 nM of N protein, with the signal intensity increasing proportionally with higher protein concentrations (Fig. 1D). These findings suggest that differences in binding affinity with regard to sequence composition and the presence of ssRNA and dsRNA, may influence the structural organization of the SARS‐CoV‐2 genome by N.

### CoV‐2 N is an RNA and DNA chaperone

To investigate the RNA chaperone activity of the SARS‐CoV‐2 nucleocapsid protein, we adopted an assay previously developed to study the chaperone function of the SARS‐CoV‐1 N protein [27].

**Fig. 2:**
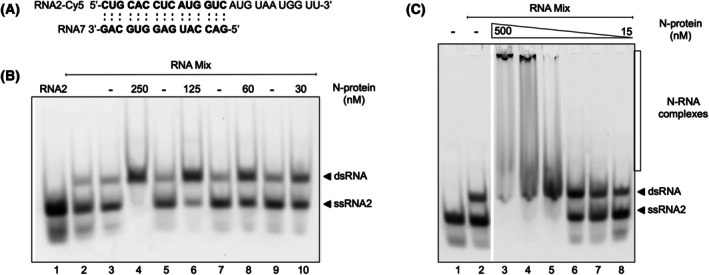
CoV‐2 N protein binds RNA and hybridizes ssRNA to dsRNA. (A) Sequences of ssRNA2 (nt 5′‐498‐520‐3′) and ssRNA7 (nt 3′‐498‐520‐5′ reverse). Complementary nucleotides for hybridization are marked bold. (B) Complementary ssRNA2‐Cy5 and ssRNA7 were mixed and incubated in the presence (indicated in Lanes 4, 6, 8, and 10), or absence (indicated in Lanes 2, 3, 5, 7, and 9) of N protein. Reactions were stopped with a sodium dodecyl sulfate (SDS) containing buffer to inactivate the protein. Samples were applied to a native 6% polyacrylamide (PAA) gel. RNA hybridization is enhanced in the presence of SARS‐CoV‐2 N protein. Lane 1 served as a control with ssRNA2‐Cy5 alone. Number of replicates *n* = 3. (C) Binding of the SARS‐CoV‐2 N protein toward hybridized RNA2 (Lane 2) was analyzed using a concentration range of 500–15 nM (Lanes 3–8) of N protein. Samples were run on a native 6% PAA TBE gel. A binding shift can be observed in the presence of N protein. Number of replicates *n* = 3.

Our assay uses two complementary ssRNAs, the 26 nt long Cy5‐labeled ssRNA2 and the 15 nt long ssRNA7 (Table S2) (Fig. 2A). The RNAs were mixed, heated to 95 °C for 2 min, and rapidly cooled on ice. Then increasing amounts of N protein were added and incubated for 3 min at RT. To stop the reaction, an SDS‐containing buffer was added to inactivate the protein, and the resulting RNA species were resolved by PAGE and visualized by fluorescence detection.

Our results revealed that the addition of CoV‐2 N protein strongly enhanced RNA hybridization in a concentration‐dependent manner. A clear annealing was already observed at protein concentrations as low as 30 nM (Lane 10). At 250 nM N protein (Lane 4), complete annealing of ssRNA to dsRNA was achieved, evident by the disappearance of the ssRNA band and a corresponding increase in dsRNA signal intensity (Fig. 2B).

These findings confirm that the SARS‐CoV‐2 N protein functions as an RNA chaperone, promoting the hybridization of ssRNA to dsRNA. To evaluate the extent of spontaneous RNA hybridization without N protein, complementary RNAs were denatured individually, rapidly cooled on ice, and only then mixed together. Under these conditions, hybridization occurred immediately upon mixing but did not increase over a 5‐min period, indicating that spontaneous annealing reaches a rapid equilibrium. Notably, the degree of hybridization did not differ from conditions where both RNAs were mixed and denatured together before cooling. In contrast, in the presence of N_WT, complete hybridization was observed within 30 s, highlighting the N proteins potent RNA chaperone ability (Fig. S2A,B). To further dissect the chaperoning mechanism, we analyzed the time‐dependency of this process using 100 nM N_WT (Fig. S2C,D). Strikingly, a substantial portion of the ssRNA had already hybridized into dsRNA after only 10 s of incubation with the protein. After approximately 180 s, no ssRNA remained in the reaction, indicating that the N‐mediated RNA chaperoning is a fast and efficient process.

To explore the relationship between RNA chaperone activity and RNA binding, we further analyzed the RNA‐binding properties of the N protein. RNA binding, and RNA hybridization occurred within comparable protein concentration ranges (Fig. 2C), indicating that efficient RNA binding is required for the chaperone activity of the protein. Even though SARS‐CoV‐2 is a ssRNA virus, the N protein also binds to DNA and can chaperone complementary ssDNA strands (Fig. S2E). However, the DNA chaperone activity is reduced compared to RNA, with detectable DNA hybridization starting at concentrations of 500 nM protein, corresponding to full DNA binding in EMSA (Fig. S2F). In summary, these findings highlight the role of the SARS‐CoV‐2 N protein in organizing and structuring the viral RNA genome, a function that may be critical for efficient viral replication and genome packaging.

### SARS‐CoV‐2 N replaces non‐perfectly matched RNA strands

As RNA folding is a highly dynamic process, RNA molecules can form intermediate structures and may become trapped in misfolded secondary configurations. RNA chaperones play a crucial role in facilitating correct folding by resolving misfolded RNAs, ultimately guiding the formation of the thermodynamically most stable state. Thus, a key characteristic of RNA chaperones is their ability to distinguish between properly folded and misfolded RNA structures, and to promote the exchange of thermodynamically unfavorable RNA conformations [15].

**Fig. 3:**
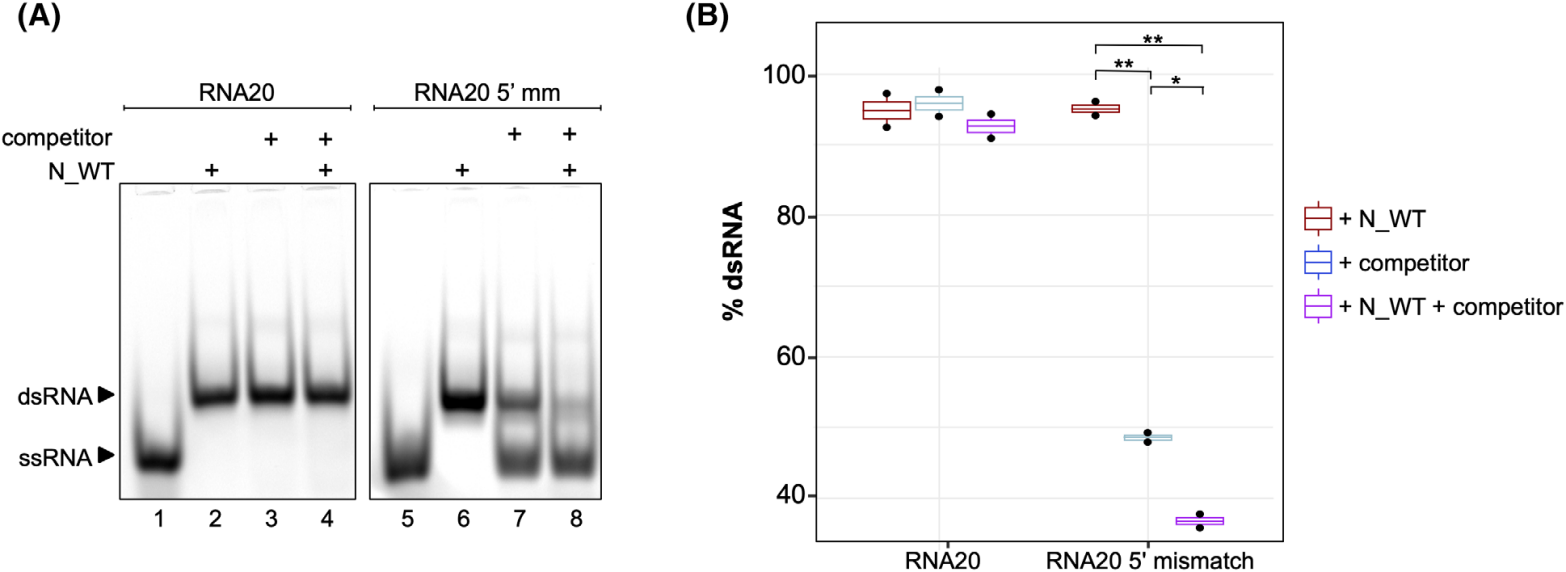
SARS‐CoV‐2 N exchanges mismatched RNA strand. (A) Strand‐exchange RNA chaperone assay: Single‐stranded (ss) RNA20‐Cy5 (Lane 1) or ssRNA20‐5′‐mismatch (Lane 5) was fully hybridized with ssRNA21 to form double‐stranded (ds) RNA complexes facilitated by wild‐type N protein (Lanes 2, 6). Competitor RNA20 was then either added directly to the dsRNA in the presence of the N protein (Lanes 4, 8), in the absence of N protein (Lane 3) or following the inactivation of N protein activity by sodium dodecyl sulfate (SDS), prior to incubation with an excess of competitor ssRNA20 (Lane 8). Whereas there is no change observable for the perfectly matching RNA20, the mismatched RNA was displaced from the double strand upon the addition of competitor RNA (Lane 7), with this removal being significantly enhanced by the presence of the N protein (lane 8). Number of replicates *n* = 3. (B) Each boxplot summarizes data from two biological replicates of the strand‐exchange RNA chaperone assays, as shown in Figure A. Additional replicates (*n* = 2) were performed at different RNA concentrations, yielding the same overall result, but could not be included in the box plot representation. The boxplots illustrate the interquartile range (IQR), with the median indicated by a horizontal line within each box. Whiskers extend to a maximum of 1.5 times the IQR above and below the upper and lower quartiles, respectively. The x‐axis categorizes the different RNA types, while the y‐axis represents the percentage of dsRNA present. Signal intensities of ssRNA and dsRNA bands were quantified using FIJI, and the percentage of dsRNA in each lane was plotted for each RNA type. The control, shown in red (+ N_WT), represents the fully hybridized RNA strand without competitor. Conditions with only the competitor are depicted in blue, and those with both the competitor and the N protein are shown in purple. No significant change is observed for RNA20, while significant differences in dsRNA integration are detectable for RNA20‐5′‐mismatch. Statistical analysis was performed by applying a *t*‐test using the Benjamini–Hochberg (BH) procedure. ‘n.s.’, not significant; *0.05 ≤ *P* > 0.01; **0.01 ≤ *P* > 0.001.

To investigate this property in the SARS‐CoV‐2 N protein, we performed an N‐mediated strand exchange assay. In this assay, we examined whether the addition of an excess of perfectly complementary competitor RNA would facilitate the exchange of already hybridized dsRNA molecules. The dsRNA molecules are either perfectly complementary or contain mismatches that were tested in the absence (Fig. 3A, Lanes 3, 7) and presence (Fig. 3A, Lanes 4, 8) of the SARS‐CoV‐2 WT N protein. Either the ssRNA20 or the ssRNA20‐5′‐mismatch RNA was hybridized with the complementary ssRNA21 molecule. Compared to ssRNA20, ssRNA20‐5′‐mismatch harbors six mismatches at its 5′ end (Table S2). Following the initial hybridization reaction in the presence of N, the protein was inactivated using an SDS‐containing stop solution before the addition of the competitor ssRNA20 (Fig. 3A, Lanes 3, 7). This control reaction served to monitor the N‐independent RNA strand exchange. In the N‐dependent exchange reaction, the protein was not inactivated prior addition of competitor RNA to the N‐containing reaction mixture (Fig. 3A, Lanes 4, 8). Additional control experiments lacking the competitor RNA reveal the complete hybridization of the dsRNA at the used N‐conditions (Fig. 3A, Lanes 2, 6).

As illustrated in Fig. 3A, Lanes 1–4, and quantified as a mean of three experiments (Fig. 3B), no exchange occurs for perfectly matching RNAs after the addition of competitor RNA. In contrast, for a mismatched dsRNA strand, the presence of a perfectly matching competitor ssRNA results in the displacement of the mismatched RNA already without functional N protein (Lane 7). However, the presence of functional N significantly enhances the substitution of the mismatched RNA (Lane 8). The data suggest that N is not capable to separate perfect double‐stranded regions but requires mismatched RNA molecules for its chaperone activity.

### The RBD‐IDR2‐CTD region of N harbors the RNA chaperone activity

**Fig. 4:**
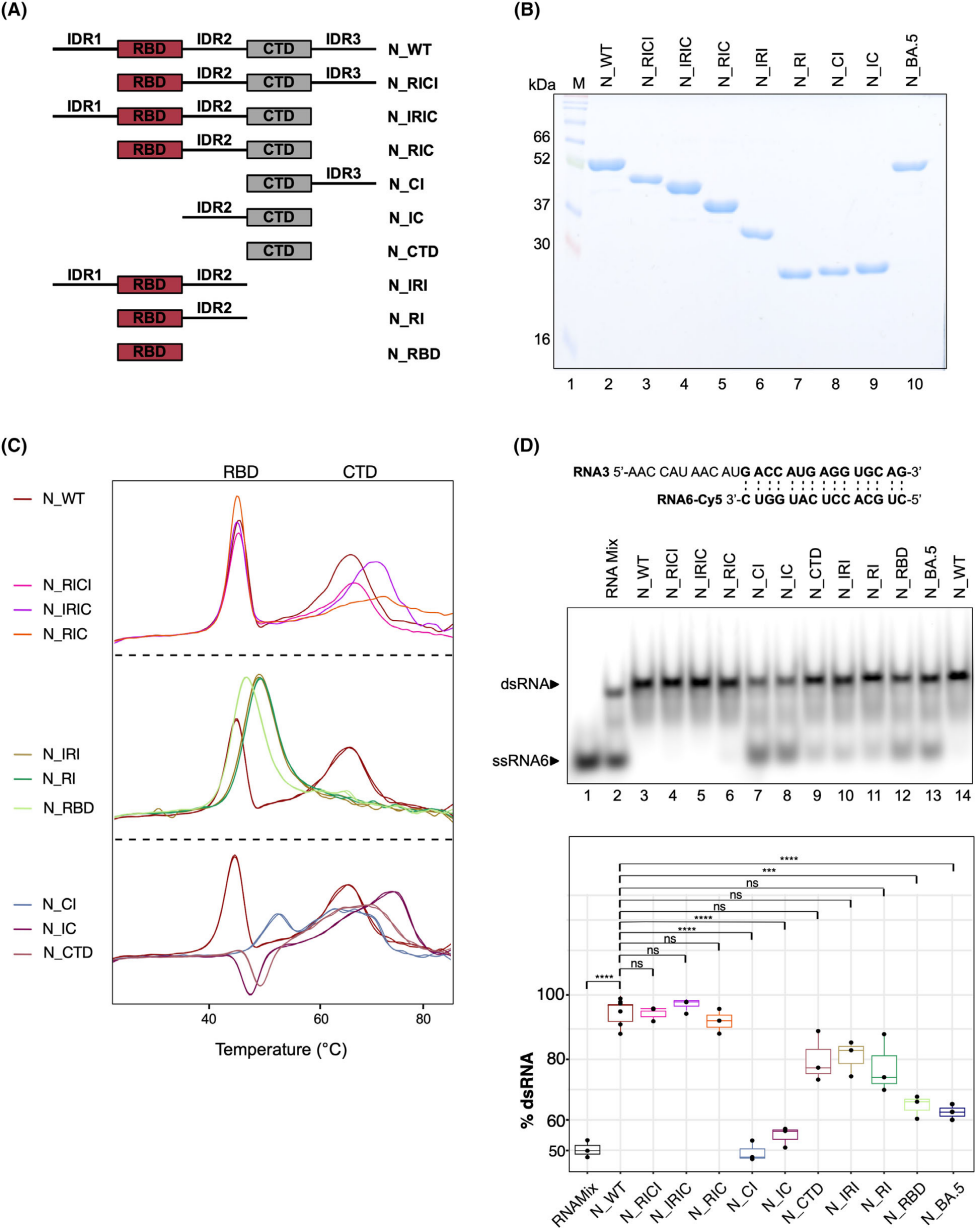
SARS‐CoV‐2 N protein truncation proteins with their respective protein stability and RNA chaperoning ability. (A) Schematic representation of the generated SARS‐CoV‐2 N protein truncation variants and the corresponding nomenclature used. (B) SDS/PAGE gel analysis of purified recombinant N protein truncations. 2 μg of each protein were loaded onto a 10% SDS/PAGE gel. Protein bands observed align with the calculated molecular weights. (C) Protein stability assessment using differential scanning fluorometry (nanoDSF) measurements. Of each protein variant, 10 μm were loaded into standard capillaries and subjected to a temperature gradient ranging from 25 °C to 85 °C using the Prometheus NT.48 nanoDSF device. Unfolding was monitored in real‐time by measuring changes in intrinsic tryptophan fluorescence at 350 nm and 330 nm. The ratio of these wavelengths and the corresponding first derivative were used to determine melting points for the folded domains of the proteins. For N_WT (wild‐type), the structured RBD (receptor‐binding domain) and CTD (C‐terminal domain) domains unfold at 45 °C and 68 °C, respectively. Truncation of specific protein regions led to alterations in unfolding behavior. (D) Boxplot depicting the percentage of hybridized dsRNA, with RNA band intensities quantified using FIJI. Each boxplot includes three biological replicates from the chaperone assay, representing the interquartile range (IQR) with the median marked by a horizontal line inside the box. Whiskers extend up to 1.5 times the IQR above and below the upper and lower quartiles, respectively. The x‐axis labels the corresponding N truncation proteins, while the y‐axis indicates the percentage of dsRNA. Significant differences in dsRNA formation were observed between the RNAMix control (black) and N_WT (dark red). Additionally, significant variations in RNA annealing efficiency relative to N_WT were noted for N_CI (gray‐blue), N_IC (dark purple), N_RBD (light green), and N_BA.5 (dark blue). Differences observed for other proteins in comparison to N_WT were statistically not significant (n.s.). Statistical analysis was conducted using a *t*‐test with the Benjamini–Hochberg (BH) procedure. Statistical significance is denoted as follows: ‘n.s.’, not significant; ***0.001 ≤ *P* > 0.0001; ****0.0001 ≤ *P*. Number of replicates *n* = 3.

For many viral RNA chaperones, disordered domains play a crucial role in the efficient folding of RNAs [8]. The SARS‐CoV‐2 N protein contains approximately 50% predicted disordered regions, including three IDRs flanking its two well‐structured domains [39]. To study the mechanism of RNA chaperoning, we generated CoV‐2 N deletion variants, lacking individual protein domains (Fig. 4A). These proteins were expressed in bacteria and purified to apparent homogeneity (Fig. 4B). The proper folding of the truncated proteins was assessed by nanoDSF (Fig. 4C). The wild‐type N protein (N_WT) showed *T*_M_ being in line with those of SARS‐CoV [40]. The isolated domains N_RBD (light green) and N_CTD (brown) each displayed a single *T*_M_ peak, with *T*_M_ values comparable to those within the full‐length wild‐type protein, indicating similar folding and stability properties of the truncated proteins. N_RICI (pink), lacking the IDR1, behaved similarly to N_WT, while N_IRIC (purple), lacking the IDR3 and N_RIC (orange), lacking IDR1 and IDR3, exhibited an increased *T*_M_ for the CTD domain, suggesting that the IDR's interact with the CTD and partially destabilize it. Truncations lacking the CTD, namely N_IRI (olive), lacking IDR3 and CTD, and N_RI (green), RBD and IDR2 only, displayed an increased *T*_M_ of the RBD domain, as compared to N_RBD and N_WT. The CTD peak of N_IC (dark purple), consisting of IDR2 and CTD, also exhibited increased stability (+5 °C), whereas N_CI (gray‐blue; CTD and IDR3), retained the *T*_M_ of isolated CTD (N_CTD). Overall, the structured domains remained folded across all N deletion variants. The observed *T*_M_ shifts indicate stronger RBD–CTD interactions in the absence of certain IDRs, leading to general structural stabilization.

To delineate which domains contribute to the RNA chaperone activity, we tested each N variant for its ability to promote RNA annealing. Complementary ssRNA3 and ssRNA6‐Cy5 were co‐incubated in equimolar protein concentrations (250 nM). Prior to comparative analysis, the RNA chaperone activity of each individual truncation protein was titrated (data not shown) to define a reaction window suitable for side‐by‐side assessment of all N variants on a single gel. As shown in the chaperone assay and the quantification from three independent replicates (Fig. 4D), significantly reduced chaperone activity was observed for N_CI, N_IC, N_CTD, N_IRI, N_RI, and N_RBD, compared to N_WT. We identified N_RIC as the minimal construct retaining the RNA chaperone function comparable to that of the full‐length protein, thereby pinpointing the RBD‐IDR2‐CTD region as essential for efficient RNA chaperoning.

Importantly, we observed that the BA.5 Omicron N protein showed markedly reduced chaperone activity compared to the Wuhan wild‐type N protein. The two variants differ by only eight amino acids and notably, only three of these amino acid exchanges (E136D, R203K, G204R) fall into the functionally active chaperone region (RBD‐IDR2‐CTD). These amino acids in the RBD and the IDR2 present a direct link between the impaired RNA annealing capability of the BA.5 N protein. This discrepancy raises the intriguing question of how the Omicron BA.5 variant achieves higher infectivity despite its reduced chaperoning activity. One possible explanation could be compensatory mechanisms elsewhere in the viral replication cycle or interactions with host factors that enhance viral fitness. Further investigation is needed to determine how these mutations influence overall viral function. In summary, we show that a combination of RBD, IDR2 and CTD is the key player in the chaperoning mechanism.

### Inhibition of phase separation does not alter N‐mediated RNA chaperoning

**Fig. 5:**
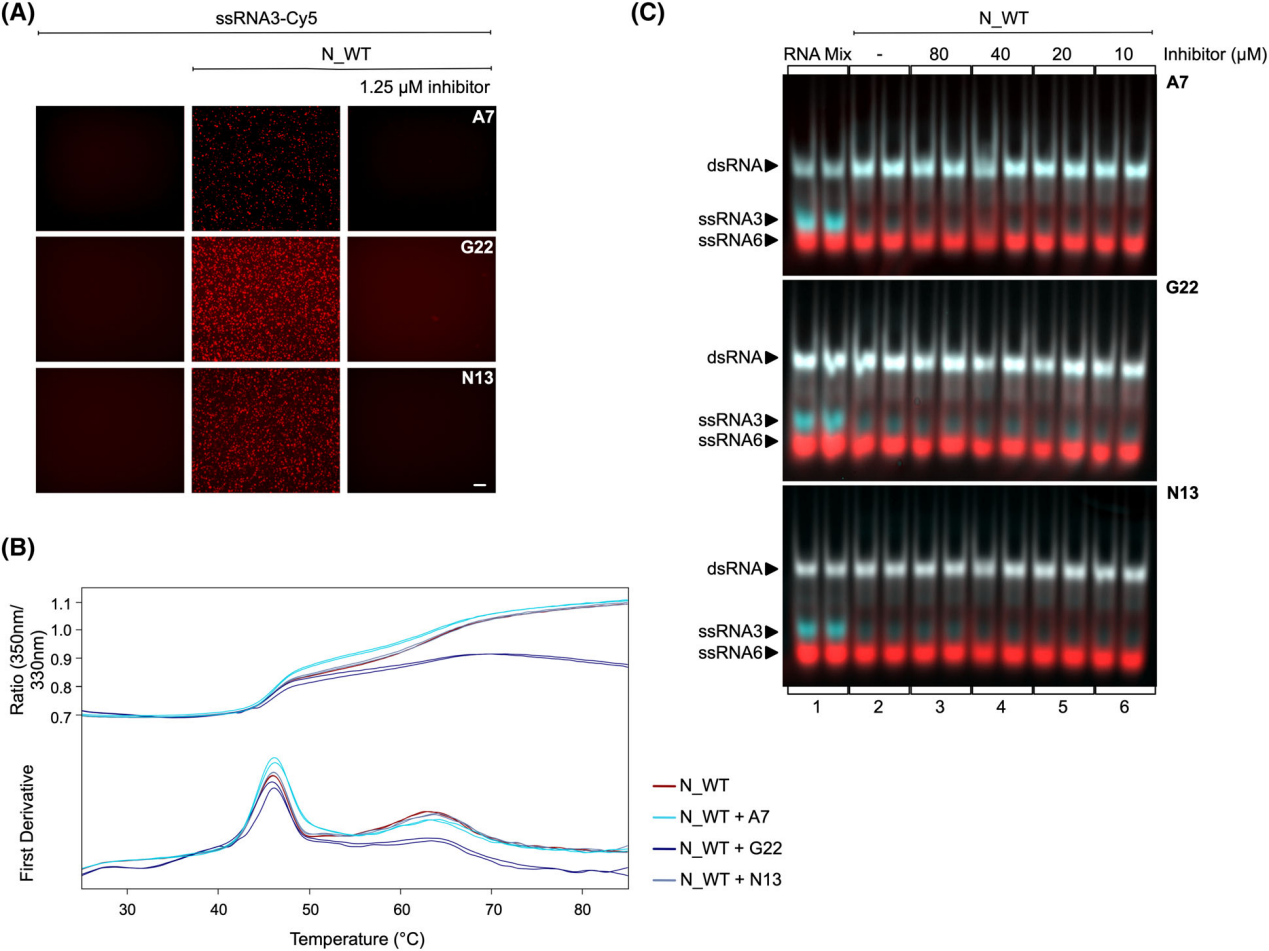
Effect of N‐RNA LLPS inhibitors on phase separation, protein stability and N‐mediated RNA chaperone activity. (A) Liquid–liquid phase separation (LLPS) experiments were conducted using 100 nM of single‐stranded RNA3 (ssRNA3) labeled with Cy5, incubated with 2 μm of N protein, in the presence or absence of 1.25 μm of inhibitors A7, G22 and N13. After a 2‐h incubation at room temperature, droplet formation was assessed by fluorescent microscopy at 40× magnification. No droplet formation is detectable in presence of A7, G22 and N13. Scale bar = 25 μm. Number of replicates *n* = 3. (B) The stability of N protein was measured in absence (dark red) and presence of the inhibitors using Differential Scanning Fluorometry (nanoDSF). A mixture of 10 μm N protein and 1.25 μm of the respective compound (A7 (light blue), G22 (dark blue), N13 (purple)) was loaded into capillaries and subjected to a temperature gradient ranging from 25 °C to 85 °C using the Prometheus NT.48 nanoDSF. Protein unfolding was monitored to assess the impact of the inhibitors on N protein stability. RBD (receptor‐binding domain) and CTD (C‐terminal domain) unfolding peaks are displayed over the temperature range. CTD peak flattens in presence of N13. (C) RNA chaperone assay in absence and presence of inhibitor compounds A7, G22 and N13. A total of 100 nM ssRNA3‐Cy3 and ssRNA6‐Cy5 were mixed with 500 nM of N protein (Lane 2) and increasing inhibitor concentrations from 10 to –80 μm (Lanes 3–6). The presence of the N protein leads to a full RNA hybridization remaining active even in the absence of phase separation. Number of replicates *n* = 3.

The correct folding of the RNA genome is likely essential for the viral infection cycle and infectivity. A detailed understanding of the SARS‐CoV‐2 N protein's chaperone mechanism and its role in RNA genome folding could facilitate the development of functional antiviral drugs. To explore this, we analyzed novel small molecules that bind to the N protein and inhibit N‐RNA LLPS, identified through a small molecule library screen (Fraunhofer ITEM, Personalized Tumor Therapy). Among the identified inhibitors, A7, G22 and N13 efficiently disrupted N‐RNA interaction‐mediated droplet formation (Fig. 5A). As described above, RNA binding is a prerequisite for efficient chaperoning. Incubation of 100 nM ssRNA3‐Cy5 with 2 μm N protein, results in efficient droplet formation in the absence of inhibitors. The presence of A7, G22 or N13 at 1.25 μm completely inhibited N–RNA interactions, effectively preventing droplet formation.

To gain insights into the specific interaction sites of these inhibitors on the N protein, we characterized the effects by nanoDSF (Fig. 5B). The inhibitors induced changes in the *T*_M_ values of N, indicating a direct interaction with the protein, without disrupting its overall folding. Next, we evaluated the ability of these LLPS inhibitors to interfere with its RNA chaperone activity (Fig. 5C). The N protein efficiently mediated RNA hybridization (Panel 2), and this activity was retained even in the presence of increasing inhibitor concentrations (Panels 3–6). As we did not observe any inhibitory effect on RNA chaperone activity with 1.25 μm inhibitor (data not shown), we used excessive inhibitor concentrations (up to 80 μm) to prove that inhibitor binding to N does not interfere with the N protein mediated RNA chaperone activity.

Even though the inhibitors A7, G22 and N13 interact with either the RBD or CTD domains required for efficient chaperoning, they do not interfere with the RNA chaperone activity. In summary, we conclude that the N‐mediated RNA chaperone mechanism operates independently of RNA tethering by LLPS.

### Strong ionic interactions reduce N‐mediated RNA hybridization

**Fig. 6:**
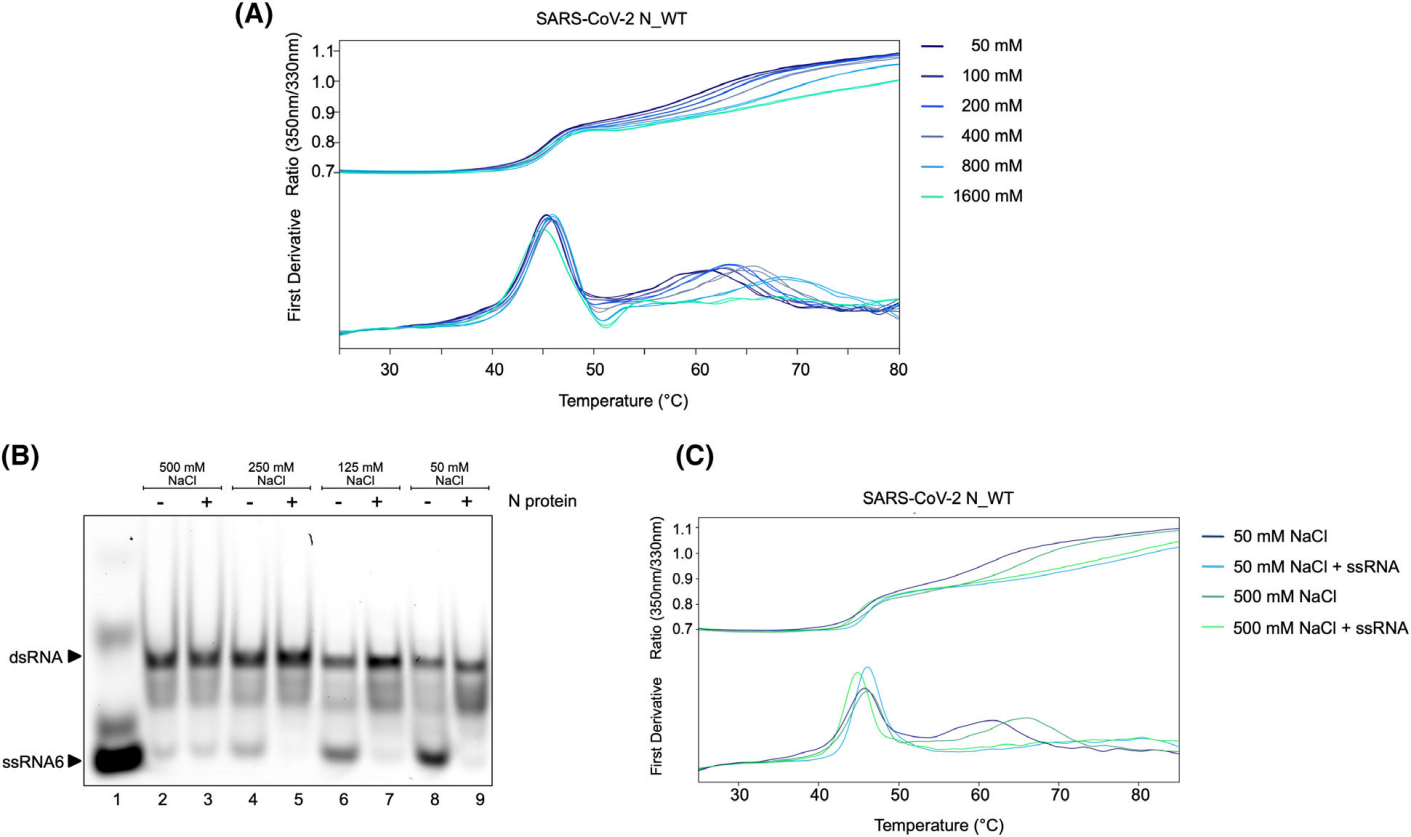
N protein‐mediated RNA chaperoning is reduced at high salt concentration. (A) Protein stability of wild‐type N protein was assessed using Differential Scanning Fluorometry (nanoDSF) with a temperature gradient ranging from 25 °C to 80 °C at different salt concentrations. 10 μm N were loaded into standard capillaries and subjected to a temperature gradient using the Prometheus NT.48 nanoDSF device. Unfolding was monitored in real‐time by measuring changes in intrinsic tryptophan fluorescence at 350 nm and 330 nm. The ratio of these wavelengths and the corresponding first derivative were used to determine melting points for the folded domains of the proteins. The stability of the C‐terminal domain (CTD) was found to be affected by high salt concentration. Number of replicates *n* = 3. (B) RNA chaperone activity of WT N protein was tested under varying salt concentrations in the reaction buffer. 100 nM RNA mix (ssRNA6‐Cy5, ssRNA3) was incubated with in absence (Lanes 2, 4, 6, 8) or presence (Lanes 3, 5, 7, 9) of 500 nM N at 50 mm (Lanes 8, 9), 125 mm (Lanes 6, 7), 250 mm (Lanes 4, 5) and 500 mm NaCl (Lanes 2, 3). ssRNA signal disappears in presence of N protein, except at 500 mm salt. Number of replicates *n* = 3. (C) N protein stability at 50 mm (blue curves) and 500 mm (green curves) salt in absence (dark blue/dark green) or presence (light blue/light green) of ssRNA was assessed. The protein binds ssRNA salt independently. Number of replicates *n* = 3.

In addition to factors like protein–protein interactions and conformational changes, other factors, like ionic interactions, may also affect the RNA chaperone activity of N [41]. To explore the influence of salt concentration and ionic strength on the chaperoning mechanism of the SARS‐CoV‐2 N protein, we first assessed protein stability at increasing salt concentrations (Fig. 6A), followed by the RNA chaperone assay performed at increasing salt concentrations (50–500 mm NaCl) (Fig. 6B).

NanoDSF analysis was employed to determine protein stability in the range from 50 to 1600 mm NaCl. As NaCl concentrations increased, we observed a stabilization of the CTD, shifting to higher *T*_M_ values. At 1600 mm NaCl, the CTD unfolding transition is no longer detectable, which could indicate either a significant stabilization or a very low cooperativity transition. This is further supported by Fig. 6C, where the CTD peak remains stable at 500 mm NaCl without ssRNA, but disappears in the presence of ssRNA, suggesting a structural rearrangement upon RNA binding.

In contrast, the stability of the RBD was only minimally affected by increasing salt concentrations, remaining stable even at the highest salt level tested (Fig. 6A). Overall, the stability and structural integrity of the protein are not affected up to a salt concentration of 0.8 m.

The chaperone assay, typically performed at 50 mm NaCl, was repeated in the presence of 125, 250 and 500 mm NaCl. Chaperone activity remained consistent between 50 and 250 mm NaCl. However, at 500 mm salt, chaperoning activity was inhibited, as indicated by the absence of any increase in dsRNA band intensity in the presence of N protein (Fig. 6B, Lanes 2, 3). Notably, increasing salt concentrations enhanced the spontaneous hybridization of the ssRNAs, yet the N protein did not further promote this effect. Interestingly, even though the RNA chaperone mechanism was inhibited at high salt concentrations, RNA binding can still be observed at 500 mm NaCl, as confirmed by nanoDSF measurements (Fig. 6C). These findings indicate that while RNA binding to N protein persists, its RNA chaperone activity is impaired at high‐salt conditions, suggesting that the chaperoning function is at least partially dependent on ionic interactions.

### Omicron BA.5 N pseudo‐phosphorylation improves its chaperoning activity

For known RNA chaperones like the RNA‐binding protein La, protein phosphorylation has been demonstrated to impact their RNA chaperone activity [42]. The presence of essential disordered regions in RNA chaperoning proteins, is often associated with the presence of phosphorylation sites [43], suggesting a potential role for phosphorylation in RNA chaperoning. Phosphorylation has indeed been implicated in modulating the RNA chaperone activity of viral proteins during viral infections [44]. In the case of SARS‐CoV‐2, for instance, GSK‐3 was shown to phosphorylate the N protein and inhibiting GSK‐3 effectively suppressed viral replication [45].

**Fig. 7:**
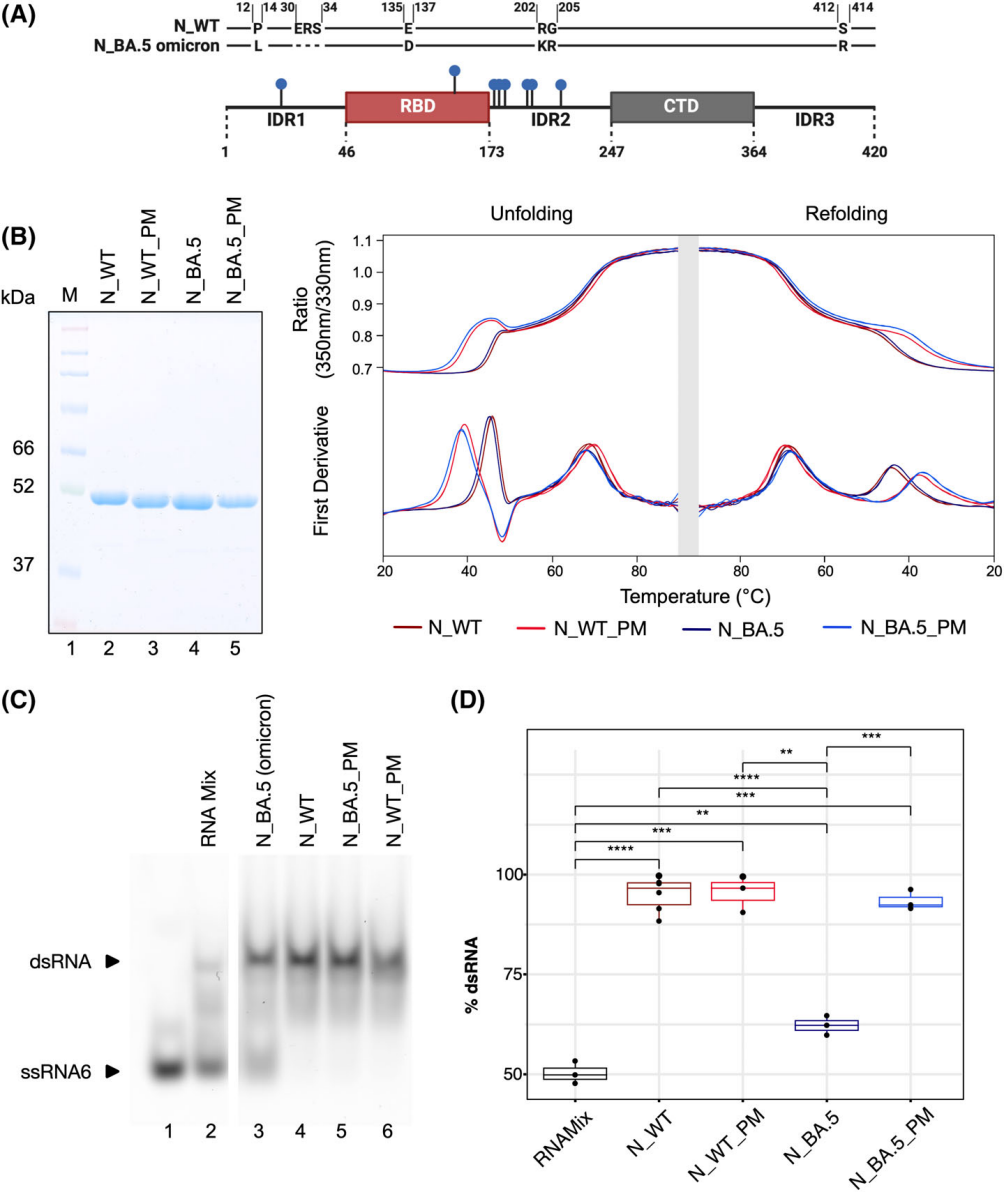
Pseudo‐phosphorylation of SARS‐CoV‐2 N leads to a decrease in stability and changed chaperone activity of BA.5 N. (A) Schematic representation of sequence differences between wild‐type N and Omicron BA.5 N (P13L, Δ31‐33, E136D, R203K, G204R, S413R) and amino acid exchanges, indicating sites of pseudo‐phosphorylation (WT N: S23, T141, S176, S180, T198, S201, S202, S206; BA.5 N: S23, T138, S173, S177, S180, T195, S198, S202, S203) by substituting serine/threonine residues with aspartic acid (blue dots). (B) Recombinant unmodified and pseudo‐phosphorylated WT and BA.5 N proteins were expressed in *E. coli* and purified using Ni‐NTA. Equal amounts (2 μg) of each protein were resolved on a 10% SDS/PAGE gel, demonstrating their molecular weights. Protein stability was evaluated using unfolding and refolding experiments conducted on a Prometheus NT.48 Differential Scanning Fluorometry (nanoDSF) device applying 10 μm of each protein, with temperature ranging from 20 °C to 90 °C. Pseudo‐phosphorylated proteins display a reduced stability of the RBD in both WT (light red) and BA.5 (light blue) N proteins. Unmodified WT (dark red) and BA.5 (dark blue) N show the expected unfolding properties. The CTD remains stable in all protein variants. Number of replicates *n* = 3. (C) Chaperone activity of unmodified and pseudo‐phosphorylated wild‐type and BA.5 N protein. RNA annealing was observed with 100 nM of ssRNA6‐Cy5 and ssRNA3 in absence (Lane 2) or presence of 250 nM N_WT (Lane 4), N_WT_PM (Lane 6), N_BA.5 (Lane 3), and N_BA.5_PM (Lane 5). Full RNA chaperone activity can be observed for N_WT, N_WT_PM, and N_BA.5_PM. A ssRNA6‐Cy5 band is visible for N_BA.5. Number of replicates *n* = 3. (D) The boxplot shows the percentage of hybridized dsRNA, with RNA band intensities analyzed using FIJI. Each boxplot represents three biological replicates from the chaperone assay, with the median indicated by a horizontal line. Whiskers extend up to 1.5 times the IQR above and below the quartiles. The x‐axis shows N truncation proteins, while the y‐axis represents dsRNA percentage. Significant differences in dsRNA formation were observed between the RNAMix control (black) and N_WT (dark red). All N variants increased dsRNA formation compared to RNAMix, with N_BA.5 (dark blue) showing the least enhancement. Each variant differed significantly from N_BA.5. N_WT_PM was similar to N_WT, while N_BA.5_PM restored the RNA chaperone activity of N_BA.5. Statistical analysis used a *t*‐test with Benjamini–Hochberg correction: **0.01 ≤ *P* > 0.001; ***0.001 ≤ *P* > 0.0001; ****0.0001 ≤ *P*.

To address the role of protein phosphorylation on the chaperone activity of the SARS‐CoV‐2 N, we generated pseudo‐phosphorylated versions of both, the WT and the BA.5 Omicron variant of the N protein. In these pseudo‐phosphorylated versions, serine or threonine residues that appeared in more than one phosphoproteomic analysis [46], were selected (WT N: S23, T141, S176, S180, T198, S201, S202, S206; BA.5 N: S23, T138, S173, S177, S180, T195, S198, S202, S203). Those amino acids were substituted by aspartic acid to mimic the phosphorylated status of the protein due to the introduction of additional negative charges. The amino acid substitutions, marked by blue dots, were primarily located within the serine‐arginine rich IDR2, with one additional substitution in each, IDR1 and RBD (Fig. 7A). Both pseudo‐phosphorylated proteins were then expressed and purified, and their intrinsic protein stability was analyzed using nanoDSF, comparing them to the unmodified proteins (N_WT dark red, N_BA.5 dark blue) (Fig. 7B).

The nanoDSF analysis revealed that pseudo‐phosphorylated WT (N_WT_PM) and BA.5 (N_BA.5_PM) N proteins displayed a lower stability of the RBD, as evidenced by a left‐shifted peak, corresponding to a *T*_M_ below 40 °C. However, the stability of the CTD remained unaffected by the pseudo‐phosphorylation (Fig. 7B).

To compare the RNA chaperone activity of both N protein variants (Wuhan and Omicron strain) and assess the impact of protein phosphorylation, complementary ssRNA3 and ssRNA6‐Cy5 were co‐incubated with equimolar amounts (250 nM) of the respective protein variants. In an initial experiment, the RNA chaperone activity was assessed for all four proteins by a titration series (Fig. S3A,B), which established a protein concentration window, suitable for the direct comparison of all proteins on a single gel.

Chaperone assays were performed using a native PAA gel (Fig. 7C), and quantitative results from three biological replicates are presented as boxplots in Fig. 7D. In the case of both N_WT (Lane 4) and N_WT_PM (Lane 6), no difference in RNA annealing activity was observed, as evidenced by complete dsRNA formation. The significantly enhanced RNA annealing activity of N_WT (dark red) and N_WT_PM (light red) relative to the RNA mix control (black), suggests that pseudo‐phosphorylation does not affect the function of the Wuhan N protein. Surprisingly, the Omicron BA.5 N protein exhibited a distinct behavior. The unmodified N_BA.5 (Lane 3) displayed reduced chaperone activity, indicated by the presence of unhybridized ssRNA. The differences in dsRNA formation between the RNAMix control (black) and N_BA.5 (dark blue), as well as between N_WT (dark red) and N_BA.5, were significant, revealing a genuine difference between these variants. Interestingly, phosphorylation of the Omicron BA.5 protein (N_BA.5_PM, Lane 5, blue) restored its RNA chaperone activity to levels comparable to the Wuhan N protein (Fig. 7C). The significant increase in dsRNA signal for N_BA.5_PM (blue) relative to the RNAMix control (black) further supports this observation.

A similar trend was observed for an additional ssRNA pair (ssRNA20/ssRNA21), originating from the SARS‐CoV‐2 5' UTR (Fig. S3C,D). This unexpected result highlights the modulatory role of phosphorylation in the RNA chaperone activity of different SARS‐CoV‐2 N protein variants, underscoring its regulatory function during viral infections.

## Discussion

Our study reveals for the first time the RNA chaperone activity of the SARS‐CoV‐2 N protein, presenting a comprehensive characterization of the specific protein domains governing this chaperoning function and the impact of N protein phosphorylation *in vitro*. The N protein plays a fundamental role in the viral life cycle, primarily responsible for genome packaging and viral assembly, while also exerting control over viral immune evasion mechanisms by blocking the host's RNA interference defenses [31, 32].

SARS‐CoV‐2 N_WT binds RNA with high affinity and displays a cooperative binding behavior, which can be attributed to the presence of two connected RNA‐binding domains. While earlier studies [33, 39, 47] have pointed to the RBD as the central element for RNA recognition, our findings show that the RBD alone does bind with low affinity (>20 μm) to the tested sequences. This discrepancy may arise from differences in experimental approaches used to assess binding. For instance, FRET and fluorescence anisotropy are highly sensitive to short‐range conformational changes or changes in molecular tumbling and can detect transient or weak interactions. In contrast, MST detects global changes in thermophoretic mobility, which primarily reflect stable complex formation under equilibrium conditions. As a result, MST may be less sensitive to transient, low‐affinity or partial interactions that do not substantially alter the physical properties of the molecule. Additionally, differences in experimental conditions, such as the use of longer or more flexible RNAs like polyU versus defined viral sequences, can significantly influence observed binding affinities and may contribute to the differences in binding efficiency reported across studies. Higher RNA‐binding affinities were obtained for the CTD, preferentially binding dsRNA, suggesting a role in genome packaging and protein dimerization. Like the CTD, N_WT exhibited preferential binding to dsRNA, despite SARS‐CoV‐2 being a ssRNA virus, reflecting the structured nature of the viral genome and the dynamic RNA conformations encountered during the viral life cycle. This capacity to recognize and interact with structured RNA forms a conceptual bridge to the N protein's function as an RNA chaperone. A substantial number of viral packaging proteins are already known to act as RNA chaperones [17], with the HIV‐1 Ncp7 protein being the most extensively studied example [25]. Furthermore, prior investigations have highlighted the RNA chaperone function of packaging proteins in coronaviruses, particularly the N protein of SARS‐CoV‐1 [27], although the underlying mechanism remained unclear. A sequence homology of 90% between SARS‐CoV‐1 and SARS‐CoV‐2 [48] led us to investigate the N protein of SARS‐CoV‐2 in this context. We show that the CoV‐2 N protein efficiently enhances the hybridization of SARS‐CoV‐2 ssRNA as well as ssDNA *in vitro*, revealing an intrinsic RNA chaperone activity. Furthermore, our assays unveiled an interdependence between RNA‐binding affinity and hybridization efficiency, where a stable RNA interaction is required for its chaperone activity. Although chaperoning requires RNA destabilization for subsequent annealing, this seemingly paradoxical function is reminiscent of the *E. coli* RNA chaperone StpA (Suppressor of td phenotype A). StpA also exhibits transient RNA binding rather than a tight RNA‐binding mode [41]. Our data support this model, as RNA annealing of the N protein was detectable even at RNA concentrations below its *K*_D_ value. Similar to StpA, whose RNA binding was shown to be highly dependent on electrostatic interactions [41], we observed that the N protein's chaperone activity is also highly sensitive to ionic strength, losing activity above 500 mm salt.

To delve deeper into the N protein‐mediated chaperone mechanism, we investigated which protein domains are required for efficient RNA annealing activity. We were able to narrow down the critical amino acid (aa) residues for the chaperone activity to the region between aa46 and aa364 (RBD‐IDR2‐CTD). This region includes the two structured domains, RBD and CTD, which are connected by the IDR2, still retaining the RNA‐binding and protein dimerization domains. IDRs have been shown to play essential roles in the activity of RNA chaperones, as they undergo cycles of unfolding and folding with their substrate [8, 16]. Previous studies highlighted the importance of the IDR1 in promoting LLPS with RNA, showing that its deletion alters the RNA concentration range required for sufficient droplet formation [49]. While LLPS plays acritical role in viral transcription, replication, cellular signaling, and stress response, we show here that the RNA chaperoning activity is independent of LLPS. Our data prove that the IDR2 of the N protein serves at least two distinct functions in the viral life cycle. While IDR1 and IDR3 of the N protein also contribute to the chaperone mechanism to some extent, deletion of these IDRs did not impair the RNA chaperone activity of the N protein. A review by Yaron *et al*. [50] highlights the regulatory role of IDRs, emphasizing their context‐dependent influence on protein function. IDRs can positively modulate domain function for additional interactions or negatively affect protein function by competing for binding partners. Our results suggest a dynamic interplay between the IDRs and the structured domains, modulating the intrinsic N protein's RNA chaperone properties. While IDRs enhance the annealing efficiency of the RBD, the addition of either IDR2 or IDR3 to the CTD reduces the chaperone activity. Conclusively, these observations indicate that, in combination with the structured RBD and CTD, the IDR2 is the major contributor to the chaperone activity of the N protein.

Interestingly, we found that the wild‐type N protein and the Omicron BA.5 variant N differ significantly in their RNA chaperone activity, with the Omicron BA.5 N exhibiting a markedly reduced RNA annealing efficiency. The protein sequences of the wild‐type and Omicron BA.5 N differ in the IDR1 (P13L, Δ31‐33), RBD (E136D), IDR2 (R203K, G204R) and IDR3 (S413R) while no mutation is present within the CTD. The amino acid substitutions in the RBD and IDR2 likely account for the reduced activity of BA.5 N. Surprisingly, we were able to restore the chaperone activity of BA.5 N to wild‐type levels by mimicking its phosphorylated state, a modification that did not impact the properties of wild‐type N. N protein phosphorylation is crucial for its function during the coronavirus infection cycle, particularly in viral genome processing [31], with SARS‐CoV‐2 taking advantage of host kinases, for example, GSK‐3 [46], for viral protein phosphorylation [51]. Especially in the early stages of SARS‐CoV‐2 infection, the serine‐/arginine‐rich IDR2 undergoes phosphorylation at multiple sites [46, 51, 52], promoting the recruitment of RNA helicase DDX1, which facilitates structural rearrangements within the RNA necessary for subgenomic RNA transcription [31]. Notably, CoV‐2 N protein phosphorylation has been shown to weaken protein–RNA interaction, thereby modulating viral ribonucleoprotein assembly [53]. To mimic the phosphorylated state, specific serine and threonine residues were substituted with aspartic acid (WT N: S23, T141, S176, S180, T198, S201, S202, S206; BA.5 N: S23, T138, S173, S177, S180, T195, S198, S202, S203), based on the phosphorylation sites identified in various phosphoproteomic analyses [46]. Both N proteins compared in our study include pseudo‐phosphorylation at identical sites, with BA.5 N containing one additional aspartic acid due to the naturally occurring E136D mutation. This additional negative charge may disrupt the intrinsic interaction between the RBD and the IDR2, potentially impairing RNA interaction and explaining the diminished chaperone activity observed for BA.5 N. Investigating whether an E136D mutation in the wild‐type N protein similarly affects RNA hybridization activity could be a compelling avenue for future research. Notably, N protein mutations found in virus variants, particularly within aa 109–205 of the IDR2, have been linked to higher viral titers and significantly increased transmissibility compared to the wild‐type [54]. The Omicron BA.5 variant, characterized by increased transmissibility and decreased pathogenicity [55], harbors two key mutations within the IDR2 (R203K, G204R), highlighting the functional importance of the aa 199–205 for efficient viral transmission. Given that these mutations reside within the chaperone‐active region of the N protein, we hypothesize a potential link between the RNA chaperone activity and viral transmissibility.

Conclusively, we identified the SARS‐CoV‐2 N protein as a multifunctional RNA‐binding protein, not only involved in viral genome packaging but also in facilitating RNA base pairing. The ability to switch between these functions appears essential during the viral infection cycle, as different genome structures are required at distinct stages of viral replication. We thus postulate that the N protein functions are tightly regulated to allow dynamic shifts in RNA‐binding behavior, supporting viral RNA replication on one hand, and assembly of newly synthesized virions on the other. This dual functionality positions the N protein, and especially its RNA chaperone activity, as a promising therapeutic target for inhibiting viral reproduction and transmission.

## Material and methods

### Plasmid constructs

The codon‐optimized DNA sequences of SARS‐CoV‐2 wild‐type nucleocapsid (N) protein (Wuhan strain) and its respective truncation sequences as well as the N protein of the Omicron BA.5 strain, which were synthesized by IDT (Integrated DNA Technologies) were cloned into the pET21a(+) expression vector (Novagen) using NdeI/XhoI restriction sites. Expressed proteins contain a C‐terminal 6×His‐Tag.

A list of the expression plasmids used in this study as well as their insert DNA and protein sequences is provided in the Table S1.

### Recombinant protein expression and purification

The SARS‐CoV‐2 nucleocapsid proteins and protein truncations were expressed using *E. coli* BL21‐AI™ One Shot™ Chemically Competent cells (Invitrogen). The bacterial cells were cultivated at 37 °C, and protein expression was induced by supplementing the growth medium with 0.5 g·L^−1^ arabinose and 1 mm IPTG when the OD_600_ reached a range of 0.5–0.8. The induced culture was incubated for 16 h at 20 °C.

Cell pellets were lysed with a denaturing lysis buffer (50 mm HEPES at pH 7.4, 500 mm NaCl, 20 mm imidazole, 6 m urea) and sonified for 5 min with 30% amplitude (Branson Ultrasonics™ Sonifier™ SFX250). The bacterial lysate was subjected to protein purification using Ni‐NTA agarose beads (Qiagen, Hilden, Germany, #30210) in accordance with the manufacturer's protocols. Washing steps were carried out at 50 mm HEPES pH 7.4, 500 mm NaCl, 20 mm imidazole, and 6 m urea. Elution steps were performed at 50 mm HEPES pH 7.4, 500 mm NaCl, 250 mm imidazole, and 6 m urea. The purified proteins were dialyzed into a buffer containing 50 mm HEPES at pH 7.4, 500 mm NaCl, and 10% glycerol. All purification steps were performed at 4 °C.

Full‐length nucleocapsid proteins (Wuhan WT and BA.5 N) were subjected to gel filtration using a Superdex 16/600 75 pg column (GE Healthcare, Düsseldorf, Germany) at buffer conditions of 50 mm HEPES pH 7.4, 500 mm NaCl, and 10% glycerol. For analysis, purified proteins (5 μg each) were separated on 10% SDS/PAGE. After analysis, purified proteins were aliquoted and stored at −80 °C.

### SARS‐CoV‐2 RNAs

ssRNA oligonucleotides of various lengths, representing viral sequences from the 5′ region, ORF1a, and the S gene of the SARS‐CoV‐2 genome, were purchased from Sigma Aldrich. RNAs were randomly chosen from the SARS‐CoV‐2 genome. RNA2, RNA3, RNA6, and RNA7 correspond to ORF1a, while RNA20, RNA20‐5′‐mm, and RNA21 originate from the 5′UTR, and RNA22 and RNA23 are derived from the S protein coding region of SARS‐CoV‐2. The complementary sequences of RNA2 and RNA3 are 26 nt long, while the complementary sequences of RNA6 and RNA7 are 15 nt long, both originating from the same genomic location. Similarly, the complementary sequences of RNA20 and RNA21, as well as RNA22 and RNA23, have the exact same length of 26 nt. A list of the RNAs used is provided in Table S2.

### Annealing of RNA oligonucleotides

Equimolar ratios (1 μm each) of unlabeled and labeled ssRNAs were mixed in annealing buffer (100 mm HEPES pH 7.4, 100 mm NaCl, 10 mm MgCl_2_). The mixture was heated to 95 °C for 2 min and allowed to slowly cool down to 25 °C. The annealed dsRNA sample was analyzed on an 8% native polyacrylamide gel and visualized using the Typhoon™ FLA 9500 (GE Healthcare, Düsseldorf, Germany) fluorescent readout.

### Protein stability measurements by nano differential scanning fluorimetry

The SARS‐CoV‐2 N protein and the respective protein truncations were analyzed by nano differential scanning fluorimetry (nanoDSF). For this, 10 μm of protein was transferred to Prometheus NT.48 Series nanoDSF Grade Standard Capillaries and analyzed using the Prometheus NT.48 device (NanoTemper Technologies, München, Germany). Measurement settings were 60–85% laser intensity, and an exemplary unfolding temperature range from 20 to 95 °C with 1 °C/min followed by a subsequent vice versa refolding ramp.

### MicroScale thermophoresis

To reveal binding affinities for the Wuhan wild‐type full‐length N protein and its distinct RBD and CTD domains toward both ssRNA and dsRNA, MicroScale thermophoresis (MST) measurements were carried out. The MST assay buffer consisted of 50 mm HEPES, 100 mm NaCl, and 0.01% Pluronic F‐127. The MST assay was set up with a 1:2 dilution series for each protein (initial concentrations: 40 μm for full‐length N protein, 80 μm for CTD, and 200 μm for RBD). Protein dilutions were mixed with 20 nM of single‐ or double‐stranded RNA3‐Cy5, RNA20‐Cy5, or RNA22‐Cy5, followed by an incubation for 30 min before loading the samples into the NT.115 Standard Capillaries (NanoTemper Technologies, München, Germany). The reactions were analyzed using the Monolith NT.115^Pico^ device (NanoTemper Technologies, München, Germany).

Measurements were performed in duplicates, each including two technical replicates. The MST experimental parameters were set as follows: 20% LED, 20% MST power, and a MST On time of 30 s at a temperature of 25 °C. Analysis of the binding affinity measurement was done at 2.5 s MST on time.

### Electromobility shift assay

20 nM Cy3‐RNA3 (ss or dsRNA) was incubated with increasing concentrations of SARS‐CoV‐2 N protein (ranging from 35 nM to 18 μm) in 0.5× PBS, 0.01% Pluronic F‐127, 5% glycerol at 25 °C for 90 min. The reaction samples were subsequently loaded onto a 1% agarose gel and electrophoresed in 1× TBE for 30 min. The RNA was detected by scanning the gel with a Typhoon™ FLA 9500 fluorescent scanner (GE Healthcare, Düsseldorf, Germany).

### RNA chaperone assay

A master mix of 100 nM ssRNA3‐Cy3 and 125 nM ssRNA6‐Cy5 (or ssRNA2 and ssRNA7, respectively) was prepared in assay buffer (10 mm Tris/HCl pH 7.6, 50 mm NaCl, 10% glycerol, 0.01% Pluronic‐F127). The RNA mix was incubated at 95 °C for 1 min and rapidly cooled down to 4 °C, ensuring the single‐stranded nature of the RNA oligos at the onset of the reaction. This procedure also applies to other complementary RNA pairs used for the chaperone assay. Afterwards, 250 nM N protein (WT, BA.5 or the respective truncation protein) was added, and the samples were incubated at 25 °C for 3 min. To stop the N‐mediated chaperoning reaction, 1 μL stop solution (20 mm Tris pH 7.6, 2.5% SDS, 25% glycerol, 50 mm EDTA) was added to each sample for protein inactivation, and samples were loaded onto 6% native polyacrylamide gels and run for 45 min at 100 V in 0.5× TBE.

### Strand‐exchange RNA chaperone assay

To assess the ability of the WT N protein to facilitate RNA strand exchange, we first generated pre‐hybridized RNA duplexes. For this, RNA master mix samples containing either ssRNA20‐Cy5 (100 nM) or ssRNA20‐5′‐mismatch‐Cy5 (100 nM) along with ssRNA21 (125/150 nM) were prepared according to the RNA Chaperone Assay protocol. Compared to ssRNA20, ssRNA20‐5′‐mismatch contains six mismatch nucleotides at its 5′ end (Table S2). Subsequently, 250 nM of wild‐type N protein was added to the mixtures and incubated for 90 min at 25 °C to ensure full hybridization of the complementary RNA molecules. The hybridized RNA complexes served as the starting condition for the assay. To test whether pre‐hybridized strands (ssRNA20, ssRNA20‐5′‐mm) could be displaced by an excess of perfectly complementary ssRNA20, we included 500 nM of this competitor RNA, using two distinct reaction conditions. For control reactions lacking active N protein: The RNA sample, which has been pre‐incubated with N protein, was treated with 1% SDS to inactivate the protein before adding competitor ssRNA20. To evaluate the activity of N protein in template exchange: Competitor ssRNA20 was added to the reaction containing the N protein and hybridized RNA. These mixtures were incubated for 2 h at 25 °C. The final reactions were stopped following the procedure described in the RNA Chaperone Assay section.

### Time‐dependent RNA chaperone assay

To investigate the kinetics of the RNA chaperone reaction mediated by the SARS‐CoV‐2 N protein, a time‐course analysis was performed. Complementary single‐stranded RNAs (100 nM RNA6‐Cy5 and 125 nM RNA3) were mixed and denatured by heating to 95 °C for 2 min, followed by rapid cooling on ice. Subsequently, 100 nM of wild‐type N protein (N_WT) was added to the RNA mixture on ice. Reactions were then incubated at room temperature (RT), and aliquots were withdrawn at 10, 20, 30, 45, 60, 120, 180, 210 and 270 s. Each reaction was immediately quenched with STOP solution (20 mm Tris pH 7.6, 2.5% SDS, 25% glycerol, 50 mm EDTA) to inactivate the protein and halt further hybridization. A 0‐s control was maintained on ice throughout the experiment to serve as a baseline. All samples were resolved on a 10% TBE polyacrylamide gel, electrophoresed at 100 V for 45 min, and visualized via fluorescence detection.

### *In vitro* liquid–liquid phase separation

The experiment was set up in duplicates using a 384‐well plate. Within each well, 2 μm of ssRNA3 or dsRNA3 was combined with a total of 100 nM of Cy5‐labeled RNA3 to visualize the formation of liquid droplets. Varying concentrations of CoV‐2 N protein (ranging from 0.25 μm to 8 μm) were added to the RNA mixture. The LLPS was carried out in LLPS buffer (20 mm Tris at pH 7.6, 0.01% Pluronic‐F127, 250 mm NaCl), with a total volume of 12 μL. Control samples containing only RNA were also included for reference.

Before proceeding with the visualization of droplet formation via fluorescent microscopy (Keyence BZ‐X800, utilizing a Cy5 filter at both 4× and 40× magnifications), the samples were incubated in the dark at room temperature for 2 h.

Furthermore, the potential impact of inhibitors on the N protein‐RNA LLPS was investigated utilizing the same *in vitro* assay approach. Inhibitors were identified in a small molecule library screen (Fraunhofer ITEM, Personalized Tumor Therapy; data not shown). The LLPS set up was kept identical, except for the inclusion of inhibitors A7 (GSK J4 HCl), G22 (NSC348884) and N13 (Cloxiquine) at a concentration of 1.25 μm. The overall DMSO concentration in each well was adjusted to 1%.

Droplet formation was analyzed by quantification of the LLPS signal via FIJI. Thereby a droplet is defined by a size range of 50–3000 pixels and a circularity range of 0.8–1.

### Quantification of RNA chaperone activity with Fiji

In order to quantify the effectiveness of strand exchange and the differences in the N (truncation‐) protein's RNA chaperone efficiency, assay gels were quantified using FIJI. The efficiency was determined by the ratio of single‐stranded to double‐stranded RNA in each reaction. For this, each gel lane was delineated with a rectangle by using the ‘Select First Lane’ and ‘Select Next Lane’ functions of FIJI. Afterwards, the function ‘Plot Lanes’ was utilized to generate histograms of the intensities. Areas corresponding to the RNA signal were selected by drawing lines and measuring the area intensity using the ‘Wand’ tracing tool. For each lane, the proportion of dsRNA intensity compared to the total RNA intensity was calculated and visualized with R.

### Data visualization with R

Data visualization was conducted using R. The R packages used included dplyr, forcats, ggplot2, ggpubr, grDevices, RcolorBrewer, and tidyverse. Visualizations were designed to illustrate key findings based on the raw data obtained from MST and EMSA approaches.

## Conflict of interest

The authors declare no conflict of interest.

## Author contributions

GL, SB and, JMS conceived and planned the experiments. SB, JMS, AL, ES, JA, SW, NS, SMR, KH, and NSG carried out the experiments. GL, SB and JMS contributed to the interpretation of the results. SB and FK carried out the analysis and statistics of the results. SB and GL took the lead in writing the manuscript. All authors provided critical feedback and helped shape the research, analysis, and manuscript.

## Supporting information


**Fig. S1.** ssRNA‐ and dsRNA‐binding capabilities of SARS‐CoV‐2 N, RBD, and CTD.
**Fig. S2.** SARS‐CoV‐2 is also a DNA chaperone and RNA chaperoning is a rapid mechanism.
**Fig. S3.** Differences in RNA chaperone activity between Wuhan and Omicron BA.5 N and their phosphomimetic variants.
**Table S1.** Expression plasmids.
**Table S2.** SARS‐CoV‐2 RNAs.

## Data Availability

The authors confirm that the data supporting the findings of this study are available within the article and its supplementary materials.
