# Supplementary material for: SARS‐CoV‐2 nucleocapsid protein variants have differential RNA chaperone activity
Source: FEBS J. 2025 Nov 20;293(9):2579–98. doi: 10.1111/febs.70329 (PMC13147315; doi:10.1111/febs.70329)
Supplement: Supplementary file 1 — Fig. S1. ssRNA‐ and dsRNA‐binding capabilities of SARS‐CoV‐2 N, RBD, and CTD. Fig. S2. SARS‐CoV‐2 is also a DNA chaperone and RNA chaperoning is a rapid mechanism. Fig. S3. Differences in RNA chaperone activity between Wuhan and Omicron BA.5 N and their phosphomimetic variants. Table S1. Expression plasmids. Table S2. SARS‐CoV‐2 RNAs. [file FEBS-293-2579-s001.pdf]

Supplementary Material

Used expression plasmids

Table S1: Expression plasmids

Plasmid name	Insert coding for
pET21a(+)_SARS-CoV-2_N_WT	Full-length SARS-CoV-2 Wuhan (wildtype) nucleocapsid protein
pET21a(+)_SARS-CoV-2_N_BA.5	Full-length SARS-CoV-2 Omicron BA.5 nucleocapsid protein
pET21a(+)_SARS-CoV-2_N_WT_PM	Phosphomimic full-length SARS-CoV-2 Wuhan (wildtype) nucleocapsid protein
pET21a(+)_SARS-CoV-2_N_BA.5_PM	Phosphomimic full-length SARS-CoV-2 Omicron BA.5 nucleocapsid protein
pET21a(+)_SARS-CoV-2_N_RICI	SARS-CoV-2 Wuhan N lacking the IDR1
pET21a(+)_SARS-CoV-2_N_IRIC	SARS-CoV-2 Wuhan N lacking the IDR3
pET21a(+)_SARS-CoV-2_N_RIC	SARS-CoV-2 Wuhan N lacking the IDR1/IDR3
pET21a(+)_SARS-CoV-2_N_IRI	SARS-CoV-2 Wuhan N lacking the CTD/IDR3
pET21a(+)_SARS-CoV-2_N_RI	SARS-CoV-2 Wuhan N lacking the IDR1/CTD/IDR3
pET21a(+)_SARS-CoV-2_N_IC	SARS-CoV-2 Wuhan N lacking the IDR1/RBD/IDR3
pET21a(+)_SARS-CoV-2_N_CI	SARS-CoV-2 Wuhan N lacking the IDR1/RBD/IDR2
pET21a(+)_SARS-CoV-2_N_RBD	SARS-CoV-2 Wuhan N RBD
pRSF-Duet1_SARS-CoV-2_N_CTD	SARS-CoV-2 Wuhan N CTD

DNA sequences of N proteins and respective protein domains (5'-3')

N_WT

ATGAGCGATAATGGTCCGCAGAATCAGCGTAATGCACCGCGTATTACCTTTGGTGGTCCGAGCGATAGCACCGGT
AGCAATCAGAATGGTGAACGTAGCGGTGCACGTAGCAAACAGCGTCGTCCGCAGGGTCTGCCGAATAATACCGC
AAGCTGGTTTACCGCACTGACCCAGCATGGTAAAGAAGATCTGAAATTTCCGCGTGGTCAGGGTGTTCCGATTA
ATACCAATAGCAGTCCGGATGATCAGATTGGTTATTATCGTCGTGCAACCCGTCGTATTCGTGGTGGTGATGGTAA
AATGAAAGATCTGAGTCCGCGTTGGTATTTCTATTATCTTGGCACCGGTCCGGAAGCAGGTCTGCCGTATGGTGC
AAATAAAGATGGTATTATTTGGGTTGCCACCGAAGGTGCACTGAATACCCCGAAAGATCATATTGGCACCCGTAA
TCCGGCAAATAATGCAGCAATTGTTCTGCAGCTGCCGCAGGGTACAACCCTGCCGAAAGGTTTTTATGCCGAAG
GTAGCCGTGGTGGTAGCCAGGCAAGCAGCCGTAGCAGCAGCCGTTCACGTAATAGTAGCCGTAATAGCACACCG
GGTAGCAGCCGTGGCACCTCACCGGCACGTATGGCAGGTAATGGCGGTGATGCAGCACTGGCACTGCTGCTGC
TGGATCGTCTGAATCAGCTGGAAAGCAAAATGAGCGGTAAAGGTCAGCAGCAACAGGGTCAGACCGTTACCAA
AAAAAGCGCAGCAGAAGCAAGCAAAAAACCGCGTCAGAAACGTACCGCAACCAAAGCATATAATGTTACCCAG
GCATTTGGTCGTCGTGGTCCGGAACAGACCCAGGGTAATTTTGGTGATCAAGAACTGATTCGTCAGGGCACCGA
TTATAAACATTGGCCTCAGATTGCACAGTTTGCACCGAGCGCAAGTGCATTTTTTGGCATGAGCCGTATTGGTAT
GGAAGTTACCCCGAGCGGCACCTGGCTGACCTATACAGGTGCAATTAAACTGGATGATAAAGATCCGAACTTCA
AGGATCAGGTGATTCTGCTGAACAAACATATCGATGCCTATAAAACATTTCCGCCTACCGAACCGAAAAAAGATA
AAAAGAAAAAGGCCGATGAAACCCAGGCACTGCCGCAGCGCCAGAAAAAACAGCAGACAGTTACCCTGCTGC
CTGCAGCAGATCTGGATGATTTTAGTAAACAGCTGCAGCAAAGCATGAGCAGCGCAGATAGCACCCAGGCACTC
GAG

N_WT_PM (phosphomimic sites marked in bold red)

ATGAGCGATAATGGTCCGCAGAATCAGCGTAATGCACCGCGTATTACCTTTGGTGGTCCGAGCGAT**GAC**ACCGGT
AGCAATCAGAATGGTGAACGTAGCGGTGCACGTAGCAAACAGCGTCGTCCGCAGGGTCTGCCGAATAATACCGC
AAGCTGGTTTACCGCACTGACCCAGCATGGTAAAGAAGATCTGAAATTTCCGCGTGGTCAGGGTGTTCCGATTA
ATACCAATAGCAGTCCGGATGATCAGATTGGTTATTATCGTCGTGCAACCCGTCGTATTCGTGGTGGTGATGGTAA
AATGAAAGATCTGAGTCCGCGTTGGTATTTCTATTATCTTGGCACCGGTCCGGAAGCAGGTCTGCCGTATGGTGC
AAATAAAGATGGTATTATTTGGGTTGCCACCGAAGGTGCACTGAAT**GAC**CCGAAAGATCATATTGGCACCCGTAA
TCCGGCAAATAATGCAGCAATTGTTCTGCAGCTGCCGCAGGGTACAACCCTGCCGAAAGGTTTTTATGCCGAAG
GT**GAC**CGTGGTGGT**GAC**CAGGCA**GAC**AGCCGTAGCAGCAGCCGTTCACGTAATAGTAGCCGTAATAGC**GAC**CC
GGGT**GACGAT**CGTGGCACC**GAC**CCGGCACGTATGGCAGGTAATGGCGGTGATGCAGCACTGGCACTGCTGCTG
CTGGATCGTCTGAATCAGCTGGAAAGCAAAATGAGCGGTAAAGGTCAGCAGCAACAGGGTCAGACCGTTACCA
AAAAAAGCGCAGCAGAAGCAAGCAAAAAACCGCGTCAGAAACGTACCGCAACCAAAGCATATAATGTTACCCA
GGCATTTGGTCGTCGTGGTCCGGAACAGACCCAGGGTAATTTTGGTGATCAAGAACTGATTCGTCAGGGCACCG
ATTATAAACATTGGCCTCAGATTGCACAGTTTGCACCGAGCGCAAGTGCATTTTTTGGCATGAGCCGTATTGGTAT
GGAAGTTACCCCGAGCGGCACCTGGCTGACCTATACAGGTGCAATTAAACTGGATGATAAAGATCCGAACTTCA
AGGATCAGGTGATTCTGCTGAACAAACATATCGATGCCTATAAAACATTTCCGCCTACCGAACCGAAAAAAGATA
AAAAGAAAAAGGCCGATGAAACCCAGGCACTGCCGCAGCGCCAGAAAAAACAGCAGACAGTTACCCTGCTGC
CTGCAGCAGATCTGGATGATTTAGTAAACAGCTGCAGCAAAGCATGAGCAGCGCAGATAGCACCCAGGCACTC
GAG

N_BA.5

ATGAGCGATAATGGTCCGCAGAATCAGCGTAATGCACTGCGTATTACCTTTGGTGGTCCGAGCGATAGCACCGGT
AGCAATCAGAATGGTGGTGCACGTAGCAAACAGCGTCGTCCGCAGGGTCTGCCGAATAATACCGCAAGCTGGTT
TACCGCACTGACCCAGCATGGTAAAGAAGATCTGAAATTTCCGCGTGGTCAGGGTGTTCCGATTAATACCAATAG
CAGTCCGGATGATCAGATTGGTTATTATCGTCGTGCAACCCGTCGTATTCGTGGTGGTGATGGTAAAATGAAAGA
TCTGAGTCCGCGTTGGTATTTCTATTATCTTGGCACCGGTCCGGAAGCAGGTCTGCCGTATGGTGCAAATAAAGA
TGGTATTATTTGGGTTGCCACCGATGGTGCACTGAATACCCCGAAAGATCATATTGGCACCCGTAATCCGGCAAAT
AATGCAGCAATTGTTCTGCAGCTGCCGCAGGGTACAACCCTGCCGAAAGGTTTTTATGCCGAAGGTAGCCGTGG
TGGTAGCCAGGCAAGCAGCCGTAGCAGCAGCCGTTCACGTAATAGTAGCCGTAATAGCACACCGGGTAGCAGC
AAACGTACCTCACCGGCACGTATGGCAGGTAATGGCGGTGATGCAGCACTGGCACTGCTGCTGCTGGATCGTCT
GAATCAGCTGGAAAGCAAAATGAGCGGTAAAGGTCAGCAGCAACAGGGTCAGACCGTTACCAAAAAAAGCGC
AGCAGAAGCAAGCAAAAAACCGCGTCAGAAACGTACCGCAACCAAAGCATATAATGTTACCCAGGCATTTGGTC
GTCGTGGTCCGGAACAGACCCAGGGTAATTTTGGTGATCAAGAACTGATTCGTCAGGGCACCGATTATAAACAT
TGGCCTCAGATTGCACAGTTTGCACCGAGCGCAAGTGCATTTTTTGGCATGAGCCGTATTGGTATGGAAGTTACC
CCGAGCGGCACCTGGCTGACCTATACAGGTGCAATTAAACTGGATGATAAAGATCCGAACTTCAAGGATCAGGT
GATTCTGCTGAACAAACATATCGATGCCTATAAAACATTTCCGCCTACCGAACCGAAAAAAGATAAAAAGAAAAA
GGCCGATGAAACCCAGGCACTGCCGCAGCGCCAGAAAAAACAGCAGACAGTTACCCTGCTGCCTGCAGCAGAT
CTGGATGATTTAGTAAACAGCTGCAGCAAAGCATGAGCCGTGCAGATAGCACCCAGGCACTCGAG

N_BA.5_PM (phosphomimic sites marked in bold red)

ATGAGCGATAATGGTCCGCAGAATCAGCGTAATGCACTGCGTATTACCTTTGGTGGTCCGAGCGAT**GAC**ACCGGT
AGCAATCAGAATGGTGGTGCACGTAGCAAACAGCGTCGTCCGCAGGGTCTGCCGAATAATACCGCAAGCTGGTT
TACCGCACTGACCCAGCATGGTAAAGAAGATCTGAAATTTCCGCGTGGTCAGGGTGTTCCGATTAATACCAATAG
CAGTCCGGATGATCAGATTGGTTATTATCGTCGTGCAACCCGTCGTATTCGTGGTGGTGATGGTAAAATGAAAGA
TCTGAGTCCGCGTTGGTATTTCTATTATCTTGGCACCGGTCCGGAAGCAGGTCTGCCGTATGGTGCAAATAAAGA
TGGTATTATTTGGGTTGCCACCGATGGTGCACTGAAT**GAC**CCGAAAGATCATATTGGCACCCGTAATCCGGCAAA
TAATGCAGCAATTGTTCTGCAGCTGCCGCAGGGTACAACCCTGCCGAAAGGTTTTTATGCCGAAGGT**GAT**CGTG
GTGGT**GAC**CAGGCA**GAC**AGCCGTAGCAGCAGCCGTTCACGTAATAGTAGCCGTAATAGC**GAC**CCGGGT**GATGA**

CAAACGTACCGATCCGGCACGTATGGCAGGTAATGGCGGTGATGCAGCACTGGCACTGCTGCTGCTGGATCGTC
TGAATCAGCTGGAAAGCAAAATGAGCGGTAAAGGTCAGCAGCAACAGGGTCAGACCGTTACCAAAAAAAGCG
CAGCAGAAGCAAGCAAAAAACCGCGTCAGAAACGTACCGCAACCAAAGCATATAATGTTACCCAGGCATTTGGT
CGTCGTGGTCCGGAACAGACCCAGGGTAATTTTGGTGATCAAGAACTGATTCGTCAGGGCACCGATTATAAACA
TTGGCCTCAGATTGCACAGTTTGCACCGAGCGCAAGTGCATTTTTTGGCATGAGCCGTATTGGTATGGAAGTTAC
CCCGAGCGGCACCTGGCTGACCTATACAGGTGCAATTAAACTGGATGATAAAGATCCGAACTTCAAGGATCAGG
TGATTCTGCTGAACAAACATATCGATGCCTATAAAACATTTCCGCCTACCGAACCGAAAAAAGATAAAAAGAAAA
AGGCCGATGAAACCCAGGCACTGCCGCAGCGCCAGAAAAAACAGCAGACAGTTACCCTGCTGCCTGCAGCAG
ATCTGGATGATTTTAGTAAACAGCTGCAGCAAAGCATGAGCCGTGCAGATAGCACCCAGGCACTCGAG

N_RICI

ATGCCGAATAATACCGCAAGCTGGTTTACCGCACTGACCCAGCATGGTAAAGAAGATCTGAAATTTCCGCGTGGT
CAGGGTGTTCCGATTAATACCAATAGCAGTCCGGATGATCAGATTGGTTATTATCGTCGTGCAACCCGTCGTATTC
GTGGTGGTGATGGTAAAATGAAAGATCTGAGTCCGCGTTGGTATTTCTATTATCTTGGCACCGGTCCGGAAGCAG
GTCTGCCGTATGGTGCAAATAAAGATGGTATTATTTGGGTTGCCACCGAAGGTGCACTGAATACCCCGAAAGATC
ATATTGGCACCCGTAATCCGGCAAATAATGCAGCAATTGTTCTGCAGCTGCCGCAGGGTACAACCCTGCCGAAAG
GTTTTTATGCCGAAGGTAGCCGTGGTGGTAGCCAGGCAAGCAGCCGTAGCAGCAGCCGTTCACGTAATAGTAGC
CGTAATAGCACACCGGGTAGCAGCCGTGGCACCTCACCGGCACGTATGGCAGGTAATGGCGGTGATGCAGCACT
GGCACTGCTGCTGCTGGATCGTCTGAATCAGCTGGAAAGCAAAATGAGCGGTAAAGGTCAGCAGCAACAGGGT
CAGACCGTTACCAAAAAAAGCGCAGCAGAAGCAAGCAAAAAACCGCGTCAGAAACGTACCGCAACCAAAGCA
TATAATGTTACCCAGGCATTTGGTCGTCGTGGTCCGGAACAGACCCAGGGTAATTTTGGTGATCAAGAACTGATT
CGTCAGGGCACCGATTATAAACATTGGCCTCAGATTGCACAGTTTGCACCGAGCGCAAGTGCATTTTTTGGCATG
AGCCGTATTGGTATGGAAGTTACCCCGAGCGGCACCTGGCTGACCTATACAGGTGCAATTAAACTGGATGATAAA
GATCCGAACTTCAAGGATCAGGTGATTCTGCTGAACAAACATATCGATGCCTATAAAACATTTCCGCCTACCGAAC
CGAAAAAAGATAAAAAGAAAAAGGCCGATGAAACCCAGGCACTGCCGCAGCGCCAGAAAAAACAGCAGACA
GTTACCCTGCTGCCTGCAGCAGATCTGGATGATTTTAGTAAACAGCTGCAGCAAAGCATGAGCAGCGCAGATAG
CACCCAGGCACTCGAG

N_IRIC

ATGAGCGATAATGGTCCGCAGAATCAGCGTAATGCACCGCGTATTACCTTTGGTGGTCCGAGCGATAGCACCGGT
AGCAATCAGAATGGTGAACGTAGCGGTGCACGTAGCAAACAGCGTCGTCCGCAGGGTCTGCCGAATAATACCGC
AAGCTGGTTTACCGCACTGACCCAGCATGGTAAAGAAGATCTGAAATTTCCGCGTGGTCAGGGTGTTCCGATTA
ATACCAATAGCAGTCCGGATGATCAGATTGGTTATTATCGTCGTGCAACCCGTCGTATTCGTGGTGGTGATGGTAA
AATGAAAGATCTGAGTCCGCGTTGGTATTTCTATTATCTTGGCACCGGTCCGGAAGCAGGTCTGCCGTATGGTGC
AAATAAAGATGGTATTATTTGGGTTGCCACCGAAGGTGCACTGAATACCCCGAAAGATCATATTGGCACCCGTAA
TCCGGCAAATAATGCAGCAATTGTTCTGCAGCTGCCGCAGGGTACAACCCTGCCGAAAGGTTTTTATGCCGAAG
GTAGCCGTGGTGGTAGCCAGGCAAGCAGCCGTAGCAGCAGCCGTTCACGTAATAGTAGCCGTAATAGCACACCG
GGTAGCAGCCGTGGCACCTCACCGGCACGTATGGCAGGTAATGGCGGTGATGCAGCACTGGCACTGCTGCTGC
TGGATCGTCTGAATCAGCTGGAAAGCAAAATGAGCGGTAAAGGTCAGCAGCAACAGGGTCAGACCGTTACCAA
AAAAAGCGCAGCAGAAGCAAGCAAAAAACCGCGTCAGAAACGTACCGCAACCAAAGCATATAATGTTACCCAG
GCATTTGGTCGTCGTGGTCCGGAACAGACCCAGGGTAATTTTGGTGATCAAGAACTGATTCGTCAGGGCACCGA
TTATAAACATTGGCCTCAGATTGCACAGTTTGCACCGAGCGCAAGTGCATTTTTTGGCATGAGCCGTATTGGTAT
GGAAGTTACCCCGAGCGGCACCTGGCTGACCTATACAGGTGCAATTAAACTGGATGATAAAGATCCGAACTTCA
AGGATCAGGTGATTCTGCTGAACAAACATATCGATGCCTATAAAACATTTCCGCCTACCGAACCGAAAAAAGATA
AAAAGAAAAAGGCCGATGAAACCCAGGCACTGCCGCAGCGCCAGAAAAAACAGCAGACAGTTACCCTGCTGC
CTGCAGCAGATCTGGATGATTTTAGTAAACAGCTGCAGCAAAGCATGAGCAGCGCAGATAGCACCCAGGCACTC
GAG

N_RIC

ATGCCGAATAATACCGCAAGCTGGTTTACCGCACTGACCCAGCATGGTAAAGAAGATCTGAAATTTCCGCGTGGT
CAGGGTGTTCCGATTAATACCAATAGCAGTCCGGATGATCAGATTGGTTATTATCGTCGTGCAACCCGTCGTATTC
GTGGTGGTGATGGTAAAATGAAAGATCTGAGTCCGCGTTGGTATTTCTATTATCTTGGCACCGGTCCGGAAGCAG
GTCTGCCGTATGGTGCAAATAAAGATGGTATTATTTGGGTTGCCACCGAAGGTGCACTGAATACCCCGAAAGATC
ATATTGGCACCCGTAATCCGGCAAATAATGCAGCAATTGTTCTGCAGCTGCCGCAGGGTACAACCCTGCCGAAAG
GTTTTTATGCCGAAGGTAGCCGTGGTGGTAGCCAGGCAAGCAGCCGTAGCAGCAGCCGTTCACGTAATAGTAGC
CGTAATAGCACACCGGGTAGCAGCCGTGGCACCTCACCGGCACGTATGGCAGGTAATGGCGGTGATGCAGCACT
GGCACTGCTGCTGCTGGATCGTCTGAATCAGCTGGAAAGCAAAATGAGCGGTAAAGGTCAGCAGCAACAGGGT
CAGACCGTTACCAAAAAAAGCGCAGCAGAAGCAAGCAAAAAACCGCGTCAGAAACGTACCGCAACCAAAGCA
TATAATGTTACCCAGGCATTTGGTCGTCGTGGTCCGGAACAGACCCAGGGTAATTTTGGTGATCAAGAACTGATT
CGTCAGGGCACCGATTATAAACATTGGCCTCAGATTGCACAGTTTGCACCGAGCGCAAGTGCATTTTTTGGCATG
AGCCGTATTGGTATGGAAGTTACCCCGAGCGGCACCTGGCTGACCTATACAGGTGCAATTAAACTGGATGATAAA
GATCCGAACTTCAAGGATCAGGTGATTCTGCTGAACAAACATATCGATGCCTATAAAACATTTCCG

N_IRI

ATGAGCGATAATGGTCCGCAGAATCAGCGTAATGCACCGCGTATTACCTTTGGTGGTCCGAGCGATAGCACCGGT
AGCAATCAGAATGGTGAACGTAGCGGTGCACGTAGCAAACAGCGTCGTCCGCAGGGTCTGCCGAATAATACCGC
AAGCTGGTTTACCGCACTGACCCAGCATGGTAAAGAAGATCTGAAATTTCCGCGTGGTCAGGGTGTTCCGATTA
ATACCAATAGCAGTCCGGATGATCAGATTGGTTATTATCGTCGTGCAACCCGTCGTATTCGTGGTGGTGATGGTAA
AATGAAAGATCTGAGTCCGCGTTGGTATTTCTATTATCTTGGCACCGGTCCGGAAGCAGGTCTGCCGTATGGTGC
AAATAAAGATGGTATTATTTGGGTTGCCACCGAAGGTGCACTGAATACCCCGAAAGATCATATTGGCACCCGTAA
TCCGGCAAATAATGCAGCAATTGTTCTGCAGCTGCCGCAGGGTACAACCCTGCCGAAAGGTTTTTATGCCGAAG
GTAGCCGTGGTGGTAGCCAGGCAAGCAGCCGTAGCAGCAGCCGTTCACGTAATAGTAGCCGTAATAGCACACCG
GGTAGCAGCCGTGGCACCTCACCGGCACGTATGGCAGGTAATGGCGGTGATGCAGCACTGGCACTGCTGCTGC
TGGATCGTCTGAATCAGCTGGAAAGCAAAATGAGCGGTAAAGGTCAGCAGCAACAGGGTCAGACCGTT

N_RI

ATGCCGAATAATACCGCAAGCTGGTTTACCGCACTGACCCAGCATGGTAAAGAAGATCTGAAATTTCCGCGTGGT
CAGGGTGTTCCGATTAATACCAATAGCAGTCCGGATGATCAGATTGGTTATTATCGTCGTGCAACCCGTCGTATTC
GTGGTGGTGATGGTAAAATGAAAGATCTGAGTCCGCGTTGGTATTTCTATTATCTTGGCACCGGTCCGGAAGCAG
GTCTGCCGTATGGTGCAAATAAAGATGGTATTATTTGGGTTGCCACCGAAGGTGCACTGAATACCCCGAAAGATC
ATATTGGCACCCGTAATCCGGCAAATAATGCAGCAATTGTTCTGCAGCTGCCGCAGGGTACAACCCTGCCGAAAG
GTTTTTATGCCGAAGGTAGCCGTGGTGGTAGCCAGGCAAGCAGCCGTAGCAGCAGCCGTTCACGTAATAGTAGC
CGTAATAGCACACCGGGTAGCAGCCGTGGCACCTCACCGGCACGTATGGCAGGTAATGGCGGTGATGCAGCACT
GGCACTGCTGCTGCTGGATCGTCTGAATCAGCTGGAAAGCAAAATGAGCGGTAAAGGTCAGCAGCAACAGGGT
CAGACCGTT

N_IC

ATGGAAGGTAGCCGTGGTGGTAGCCAGGCAAGCAGCCGTAGCAGCAGCCGTTCACGTAATAGTAGCCGTAATA
GCACACCGGGTAGCAGCCGTGGCACCTCACCGGCACGTATGGCAGGTAATGGCGGTGATGCAGCACTGGCACT
GCTGCTGCTGGATCGTCTGAATCAGCTGGAAAGCAAAATGAGCGGTAAAGGTCAGCAGCAACAGGGTCAGACC
GTTACCAAAAAAAGCGCAGCAGAAGCAAGCAAAAAACCGCGTCAGAAACGTACCGCAACCAAAGCATATAATG
TTACCCAGGCATTTGGTCGTCGTGGTCCGGAACAGACCCAGGGTAATTTTGGTGATCAAGAACTGATTCGTCAG
GGCACCGATTATAAACATTGGCCTCAGATTGCACAGTTTGCACCGAGCGCAAGTGCATTTTTTGGCATGAGCCGT
ATTGGTATGGAAGTTACCCCGAGCGGCACCTGGCTGACCTATACAGGTGCAATTAAACTGGATGATAAAGATCCG
AACTTCAAGGATCAGGTGATTCTGCTGAACAAACATATCGATGCCTATAAAACATTTCCG

N_CI

ATGACCAAAAAAAGCGCAGCAGAAGCAAGCAAAAAACCGCGTCAGAAACGTACCGCAACCAAAGCATATAATG
TTACCCAGGCATTTGGTCGTCGTGGTCCGGAACAGACCCAGGGTAATTTTGGTGATCAAGAACTGATTCGTCAG
GGCACCGATTATAAACATTGGCCTCAGATTGCACAGTTTGCACCGAGCGCAAGTGCATTTTTTGGCATGAGCCGT
ATTGGTATGGAAGTTACCCCGAGCGGCACCTGGCTGACCTATACAGGTGCAATTAAACTGGATGATAAAGATCCG
AACTTCAAGGATCAGGTGATTCTGCTGAACAAACATATCGATGCCTATAAAACATTTCCGCCTACCGAACCGAAA
AAAGATAAAAAGAAAAAGGCCGATGAAACCCAGGCACTGCCGCAGCGCCAGAAAAAACAGCAGACAGTTACC
CTGCTGCCTGCAGCAGATCTGGATGATTTTAGTAAACAGCTGCAGCAAAGCATGAGCAGCGCAGATAGCACCCA
GGCACTCGAG

N_RBD

ATGCCGAATAATACCGCAAGCTGGTTTACCGCACTGACCCAGCATGGTAAAGAAGATCTGAAATTTCCGCGTGGT
CAGGGTGTTCCGATTAATACCAATAGCAGTCCGGATGATCAGATTGGTTATTATCGTCGTGCAACCCGTCGTATTC
GTGGTGGTGATGGTAAAATGAAAGATCTGAGTCCGCGTTGGTATTTCTATTATCTTGGCACCGGTCCGGAAGCAG
GTCTGCCGTATGGTGCAAATAAAGATGGTATTATTTGGGTTGCCACCGAAGGTGCACTGAATACCCCGAAAGATC
ATATTGGCACCCGTAATCCGGCAAATAATGCAGCAATTGTTCTGCAGCTGCCGCAGGGTACAACCCTGCCGAAAG
GTTTTTATGCC

N_CTD

ATGACCAAAAAAAGCGCAGCAGAAGCAAGCAAAAAACCGCGTCAGAAACGTACCGCAACCAAAGCATATAATG
TTACCCAGGCATTTGGTCGTCGTGGTCCGGAACAGACCCAGGGTAATTTTGGTGATCAAGAACTGATTCGTCAG
GGCACCGATTATAAACATTGGCCTCAGATTGCACAGTTTGCACCGAGCGCAAGTGCATTTTTTGGCATGAGCCGT
ATTGGTATGGAAGTTACCCCGAGCGGCACCTGGCTGACCTATACAGGTGCAATTAAACTGGATGATAAAGATCCG
AACTTCAAGGATCAGGTGATTCTGCTGAACAAACATATCGATGCCTATAAAACATTTCCG

Protein sequences of N protein and respective protein domains (N_{term} – C_{term})

N_WT

MSDNGPQNQRNAPRITFGGPSDSTGSNQNGERSGARSKQRRPQGLPNNTASWFTALTQHGKEDLKFPRGQGVPI
NTNSSPDDQIGYYRRATRRIRGGDGKMKDLSPRWYFYYLGTGPEAGLPYGANKDGIIWVATEGALNTPKDHIGTRN
PANNAAIVLQLPQGTTLPKGFYAEGSRGGSQASSRSSSRSRNSSRNSTPGSSRGTSPARMAGNGGDAALALLLLDRL
NQLESKMSGKGQQQQGQTVTKKSAAEASKKPRQKRTATKAYNVTQAFGRRGPEQTQGNFGDQELIRQGTDYKH
WPQIAQFAPSASAFFGMSRIGMEVTPSGTWLTYTGAIKLDDKDPNFKDQVILLNKHIDAYKTFPPTEPKKDKKKKAD
ETQALPQRQKKQQTVTLLPAADLDDFSKQLQQSMSSADSTQALE

N_WT_PM (phosphomimic sites marked in bold red)

MSDNGPQNQRNAPRITFGGPSD**D**TGSNQNGERSGARSKQRRPQGLPNNTASWFTALTQHGKEDLKFPRGQGVP
INTNSSPDDQIGYYRRATRRIRGGDGKMKDLSPRWYFYYLGTGPEAGLPYGANKDGIIWVATEGALN**D**PKDHIGTR
NPANNAAIVLQLPQGTTLPKGFYAEG**D**RGG**D**QA**D**SRSSSRSRNSSRNS**D**PG**DD**RGT**D**PARMAGNGGDAALALLL
LDRLNQLESKMSGKGQQQQGQTVTKKSAAEASKKPRQKRTATKAYNVTQAFGRRGPEQTQGNFGDQELIRQGTD
YKHWPQIAQFAPSASAFFGMSRIGMEVTPSGTWLTYTGAIKLDDKDPNFKDQVILLNKHIDAYKTFPPTEPKKDKKK
KADETQALPQRQKKQQTVTLLPAADLDDFSKQLQQSMSSADSTQALE

N_BA.5

MSDNGPQNQRNALRITFGGPSDSTGSNQNGGARSKQRRPQGLPNNTASWFTALTQHGKEDLKFPRGQGVPINT
NSSPDDQIGYYRRATRRIRGGDGKMKDLSPRWYFYYLGTGPEAGLPYGANKDGIIWVATDGALNTPKDHIGTRNPA
NNAAIVLQLPQGTTLPKGFYAEGSRGGSQASSRSSSRSRNSSRNSTPGSSKRTSPARMAGNGGDAALALLLLDRLN
QLESKMSGKGQQQQGQTVTKKSAAEASKKPRQKRTATKAYNVTQAFGRRGPEQTQGNFGDQELIRQGTDYKHW
PQIAQFAPSASAFFGMSRIGMEVTPSGTWLTYTGAIKLDDKDPNFKDQVILLNKHIDAYKTFPPTEPKKDKKKKADE
TQALPQRQKKQQTVTLLPAADLDDFSKQLQQSMSRADSTQALE

N_BA.5_PM (phosphomimic sites marked in bold red)

MSDNGPQNQRNALRITFGGPSD**D**TGSNQNGGARSKQRRPQGLPNNTASWFTALTQHGKEDLKFPRGQGVPINT
NSSPDDQIGYYRRATRRIRGGDGKMKDLSPRWYFYYLGTGPEAGLPYGANKDGIIWVATDGALN**D**PKDHIGTRNP
ANNAAIVLQLPQGTTLPKGFYAEG**D**RGG**D**QA**D**SRSSSRSRNSSRNS**D**PG**DD**KRT**D**PARMAGNGGDAALALLLLD
RLNQLESKMSGKGQQQQGQTVTKKSAAEASKKPRQKRTATKAYNVTQAFGRRGPEQTQGNFGDQELIRQGTDYK
HWPQIAQFAPSASAFFGMSRIGMEVTPSGTWLTYTGAIKLDDKDPNFKDQVILLNKHIDAYKTFPPTEPKKDKKKKA
DETQALPQRQKKQQTVTLLPAADLDDFSKQLQQSMSRADSTQALE

N_RICI

MPNNTASWFTALTQHGKEDLKFPRGQGVPINTNSSPDDQIGYYRRATRRIRGGDGKMKDLSPRWYFYYLGTGPEA
GLPYGANKDGIIWVATEGALNTPKDHIGTRNPANNAAIVLQLPQGTTLPKGFYAEGSRGGSQASSRSSSRSRNSSRN
STPGSSRGTSPARMAGNGGDAALALLLLDRLNQLESKMSGKGQQQQGQTVTKKSAAEASKKPRQKRTATKAYNVT
QAFGRRGPEQTQGNFGDQELIRQGTDYKHWPQIAQFAPSASAFFGMSRIGMEVTPSGTWLTYTGAIKLDDKDPN
FKDQVILLNKHIDAYKTFPPTEPKKDKKKKADETQALPQRQKKQQTVTLLPAADLDDFSKQLQQSMSSADSTQALE

N_IRIC

MSDNGPQNQRNAPRITFGGPSDSTGSNQNGERSGARSKQRRPQGLPNNTASWFTALTQHGKEDLKFPRGQGVPI
NTNSSPDDQIGYYRRATRRIRGGDGKMKDLSPRWYFYYLGTGPEAGLPYGANKDGIIWVATEGALNTPKDHIGTRN
PANNAAIVLQLPQGTTLPKGFYAEGSRGGSQASSRSSSRSRNSSRNSTPGSSRGTSPARMAGNGGDAALALLLLDRL
NQLESKMSGKGQQQQGQTVTKKSAAEASKKPRQKRTATKAYNVTQAFGRRGPEQTQGNFGDQELIRQGTDYKH
WPQIAQFAPSASAFFGMSRIGMEVTPSGTWLTYTGAIKLDDKDPNFKDQVILLNKHIDAYKTFPPTEPKKDKKKKAD
ETQALPQRQKKQQTVTLLPAADLDDFSKQLQQSMSSADSTQALE

N_RIC

MPNNTASWFTALTQHGKEDLKFPRGQGVPINTNSSPDDQIGYYRRATRRIRGGDGKMKDLSPRWYFYYLGTGPEA
GLPYGANKDGIIWVATEGALNTPKDHIGTRNPANNAAIVLQLPQGTTLPKGFYAEGSRGGSQASSRSSSRSRNSSRN
STPGSSRGTSPARMAGNGGDAALALLLLDRLNQLESKMSGKGQQQQGQTVTKKSAAEASKKPRQKRTATKAYNVT
QAFGRRGPEQTQGNFGDQELIRQGTDYKHWPQIAQFAPSASAFFGMSRIGMEVTPSGTWLTYTGAIKLDDKDPN
FKDQVILLNKHIDAYKTFP

N_IRI

MSDNGPQNQRNAPRITFGGPSDSTGSNQNGERSGARSKQRRPQGLPNNTASWFTALTQHGKEDLKFPRGQGVPI
NTNSSPDDQIGYYRRATRRIRGGDGKMKDLSPRWYFYYLGTGPEAGLPYGANKDGIIWVATEGALNTPKDHIGTRN
PANNAAIVLQLPQGTTLPKGFYAEGSRGGSQASSRSSSRSRNSSRNSTPGSSRGTSPARMAGNGGDAALALLLLDRL
NQLESKMSGKGQQQQGQTV

N_RI

MPNNTASWFTALTQHGKEDLKFPRGQGVPINTNSSPDDQIGYYRRATRRIRGGDGKMKDLSPRWYFYYLGTGPEA
GLPYGANKDGIIWVATEGALNTPKDHIGTRNPANNAAIVLQLPQGTTLPKGFYAEGSRGGSQASSRSSSRSRNSSRN
STPGSSRGTSPARMAGNGGDAALALLLLDRLNQLESKMSGKGQQQQGQTV

N_IC

MEGSRGGSQASSRSSSRSRNSSRNSTPGSSRGTSPARMAGNGGDAALALLLLDRLNQLESKMSGKGQQQQGQTV
TKKSAAEASKKPRQKRTATKAYNVTQAFGRRGPEQTQGNFGDQELIRQGTDYKHWPQIAQFAPSASAFFGMSRIG
MEVTPSGTWLTYTGAIKLDDKDPNFKDQVILLNKHIDAYKTFP

N_CI

MTKKSAAEASKKPRQKRTATKAYNVTQAFGRRGPEQTQGNFGDQELIRQGTDYKHWPQIAQFAPSASAFFGMSRI
GMEVTPSGTWLTYTGAIKLDDKDPNFKDQVILLNKHIDAYKTFPPTEPKKDKKKKADETQALPQRQKKQQTVTLLPA
ADLDDFSKQLQQSMSSADSTQALE

N_RBD

MPNNTASWFTALTQHGKEDLKFPRGQGVPINTNSSPDDQIGYYRRATRRIRGGDGKMKDLSPRWYFYYLGTGPEA
GLPYGANKDGIIWVATEGALNTPKDHIGTRNPANNAAIVLQLPQGTTLPKGFYA

N_CTD

MTKKSAAEASKKPRQKRTATKAYNVTQAFGRRGPEQTQGNFGDQELIRQGTDYKHWPQIAQFAPSASAFFGMSRI
GMEVTPSGTWLTYTGAIKLDDKDPNFKDQVILLNKHIDAYKTFP

RNA sequences

Table S2: SARS-CoV-2 RNAs

RNA Name	Oligonucleotide sequence 5'→3'	Position in SARS-CoV-2 genome
RNA2	CUG CAC CUC AUG GUC AUG UUA UGG UU	498 - 520
RNA3	AAC CAU AAC AUG ACC AUG AGG UGC AG	498 – 520 (reverse)
RNA6	CUG CAC CUC AUG GUC	498 - 512
RNA7	GAC CAU GAG GUG CAG	498 – 512 (reverse)
RNA20	UUC GUC CGG GUG UGA CCG AAA GGU AA	239 - 264
RNA20-5'-mismatch (mm)	**ACA UAG** CGG GUG UGA CCG AAA GGU AA	239-264
RNA21	UUA CCU UUC GGU CAC ACC CGG ACG AA	239 – 264 (reverse)
RNA22	CAU AAU AAG AGG CUG GAU UUU UGG UA	21859 - 21884
RNA23	UAC CAA AAA UCC AGC CUC UUA UUA UG-	21859 – 21884 (reverse)

Supplementary Figures

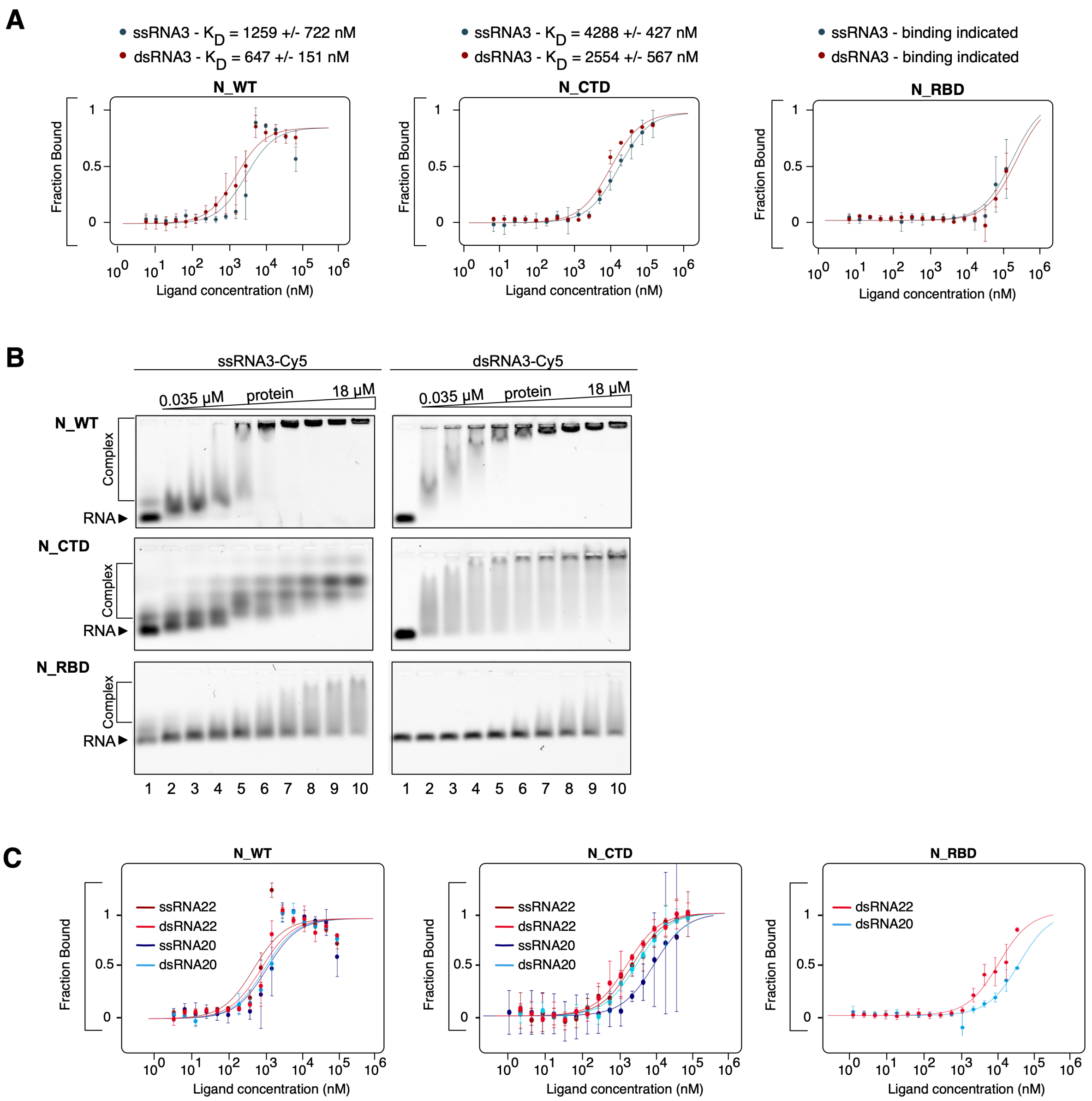

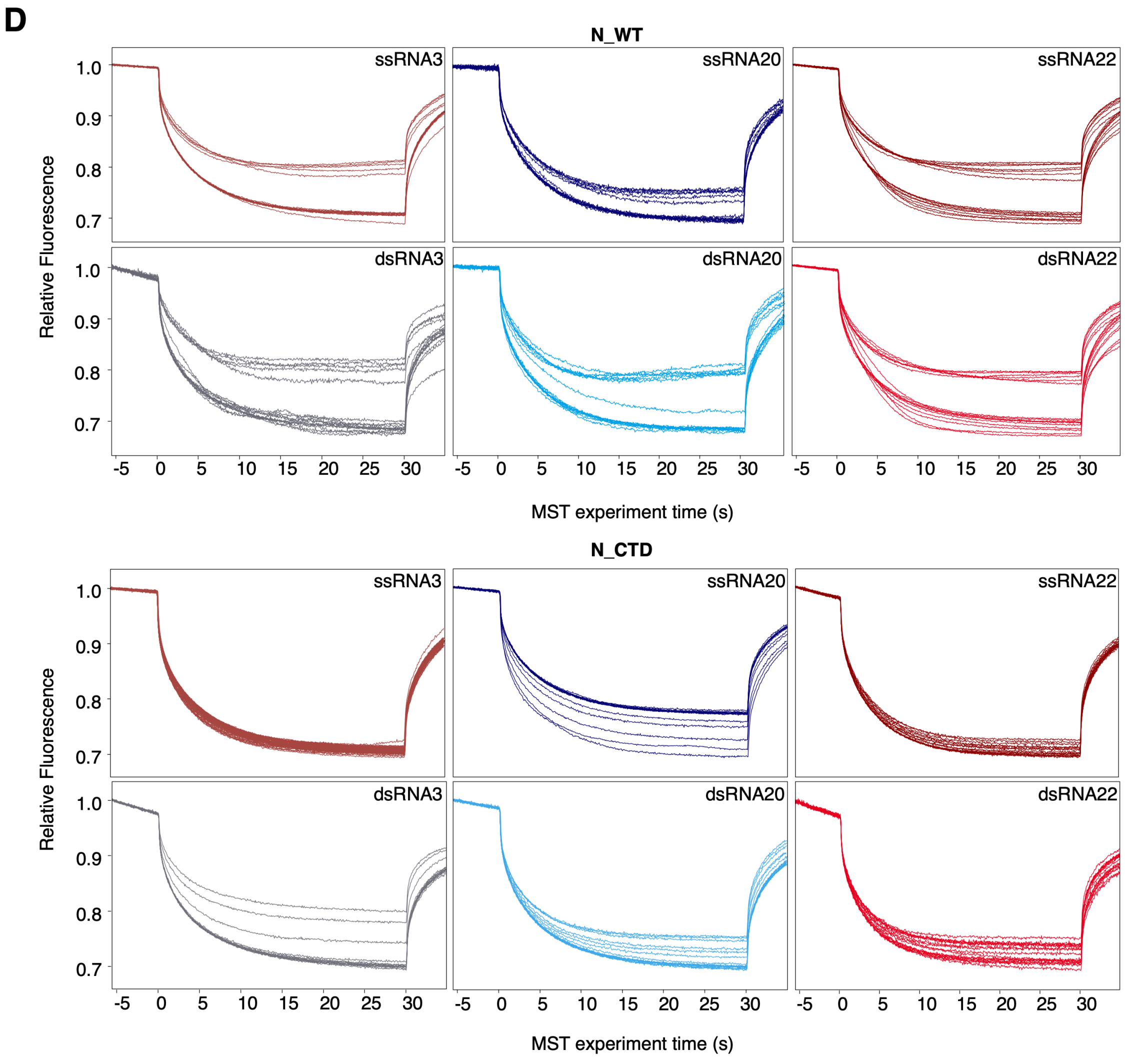

Supplementary Figure 1: ssRNA and dsRNA binding capabilities of SARS-CoV-2 N, RBD, and CTD

(A) The binding affinities of N_WT, N_CTD, and N_RBD to ssRNA3 (blue-grey) or dsRNA3 (red) were measured using Microscale Thermophoresis (MST). The MST traces, along with binding affinities (K_D) values and confidence intervals, are shown in the figure. The plots display protein concentration on the x-axis and fraction bound on the y-axis. For N_WT, K_D values were 1259 ± 722 nM for ssRNA3 and 647 ± 151 nM for dsRNA3. For N_CTD, K_D values were 4288 ± 427 nM for ssRNA3 and 2554 ± 567 nM for dsRNA3, although binding was only indicated. Number of replicates n=3.

(B) The RNA binding properties of N_WT, N_CTD, and N_RBD to ssRNA3 and dsRNA3 were further examined using electromobility shift assays (EMSA). A constant 40 nM of Cy5-labeled ssRNA3 or dsRNA3 was mixed with varying protein concentrations (0.035–18 µM) and incubated for 90 minutes. N_WT and N_CTD efficiently bound both ssRNA3 and dsRNA3, with a slight preference for dsRNA. The RBD domain exhibited weak RNA binding. Number of replicates n=3. **(C)** MST measurements to determine the binding affinities of N_WT, N_CTD, and N_RBD to ssRNA20 (dark blue), dsRNA20 (light blue), ssRNA22 (dark red), and dsRNA22 (light red). The plots display protein concentration on the x-axis and fraction bound on the y-axis. For N_WT, the fitted K_D values were 425 ± 186 nM (ssRNA20), 398 ± 157 nM (dsRNA20), 291 ± 121 nM (ssRNA22), and 344 ± 132 nM (dsRNA22). For N_CTD, K_D values were 8535 ± 4510 nM (ssRNA20), 3300 ± 394 nM (dsRNA20), 2731 ± 588 nM (ssRNA22), and 1845 ± 252 nM (dsRNA22). For N_RBD, binding could only be fitted for dsRNA. Number of replicates n=3. **(D)** Exemplary raw MST traces for N_WT and N_CTD interaction with ssRNA3 (brown), dsRNA3 (grey), ssRNA20 (dark blue), dsRNA20 (light blue), ssRNA22 (dark red), and dsRNA22 (light red). Relative fluorescence, given on the y-axis, spans from 0.7 – 1. MST experiment time (IR laser on), given on the x-axis, spans from 0 – 30s.

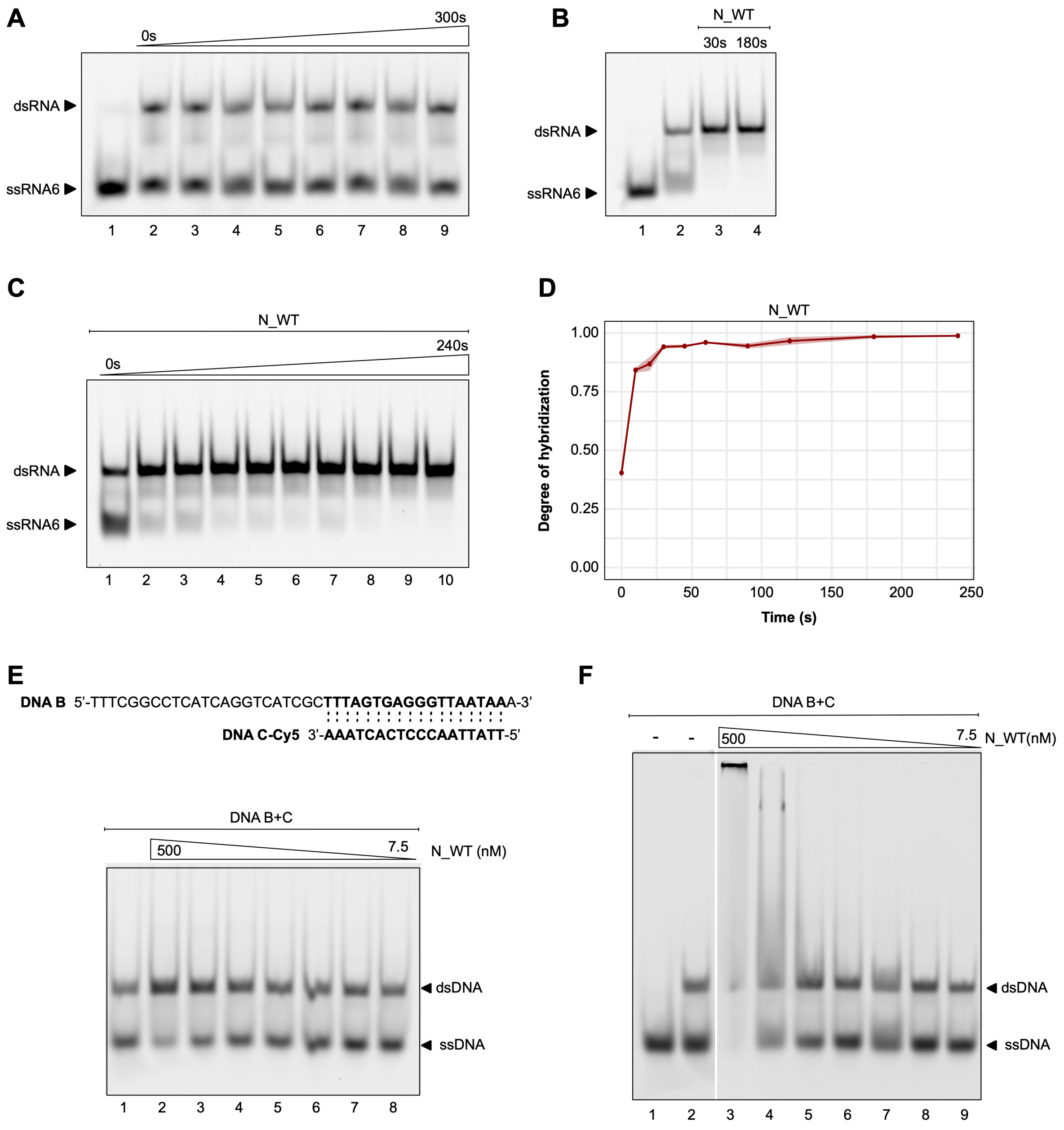

Supplementary Figure 2: SARS-CoV-2 is also a DNA chaperone and RNA chaperoning is an rapid mechanism

(A) Time-dependent RNA hybridization without protein. ssRNA6-Cy5 and ssRNA3-Cy3 were heated up to 95°C for 2 minutes individually, cooled down to 4°C. Afterwards, ssRNAs were mixed to reach final concentrations of 100 nM ssRNA6-Cy5 and 125 nM ssRNA3-Cy3 and placed to room temperature. Reactions were stopped in between 0 and 300 seconds and the degree of hybridization was visualized on a 10% native TBE gel. The 0 seconds sample (lane 2) represents the state directly after mixing. Partial hybridization occurs already directly after mixing without a substantial increase within 300 seconds (lanes 3-9). Number of replicates n=3. **(B)** Hybridization of complementary ssRNA6 and ssRNA3 in absence and presence of N-protein. As in (F), ssRNA6 and ssRNA3 were heated to 95°C for 2 minutes individually, cooled down to 4°C and then mixed to a final concentration of 100 nM ssRNA6 and 125 nM ssRNA3. Lane 2 represents the hybridization of ssRNAs without protein after 3 minutes of incubation at room temperature. Lane 3 represents RNA hybridization in presence of 250 nM N_WT for 30 seconds at room temperature and lane 4 for 3 minutes at room temperature. The N protein enhances the annealing

of the complementary RNAs. Number of replicates n=3. **(C)** Time-dependent RNA chaperone activity of N_WT. 100 nM ssRNA6-Cy5 was mixed with 125 nM ssRNA3, heated up to 95°C prior addition of 100 nM N_WT. RNA annealing was stopped after various time points between 0 – 240 seconds and visualized by fluorescence detection. The 0s sample represents the baseline, and strong RNA hybridization occurs already after 10 seconds (lane 2), with complete RNA annealing at approximately 180 seconds (lane 9). Number of replicates n=3. **(D)** Quantification of the time-dependent RNA annealing in (C). Degree of hybridization is given on the y-axis and time in seconds on the x-axis. Number of replicates n=3. **(E)** DNA hybridization activity of N_WT. Complementary Sequences of ssDNA-B and ssDNA-C. Complementary nucleotides for hybridization are marked bold. ssDNA-B and ssDNA-C-Cy5 were mixed, promoting spontaneous DNA hybridization, as evidenced in lane 1. Subsequently, CoV-2 N protein was added to the RNA Mix at concentrations ranging from 500 nM (lane 2) to 7.5 nM (lane 8). Little DNA hybridization efficiency can be observed with ssDNA started being hybridized in presence of 500 nM of N_WT. **(F)** Binding of the SARS-CoV-2 N protein towards hybridized DNA (lane 2) was analyzed using a concentration range of 7.5 – 500 nM of protein. With increasing protein amount, the DNA gets fully bound by N (lane 4). Number of replicates n=3.

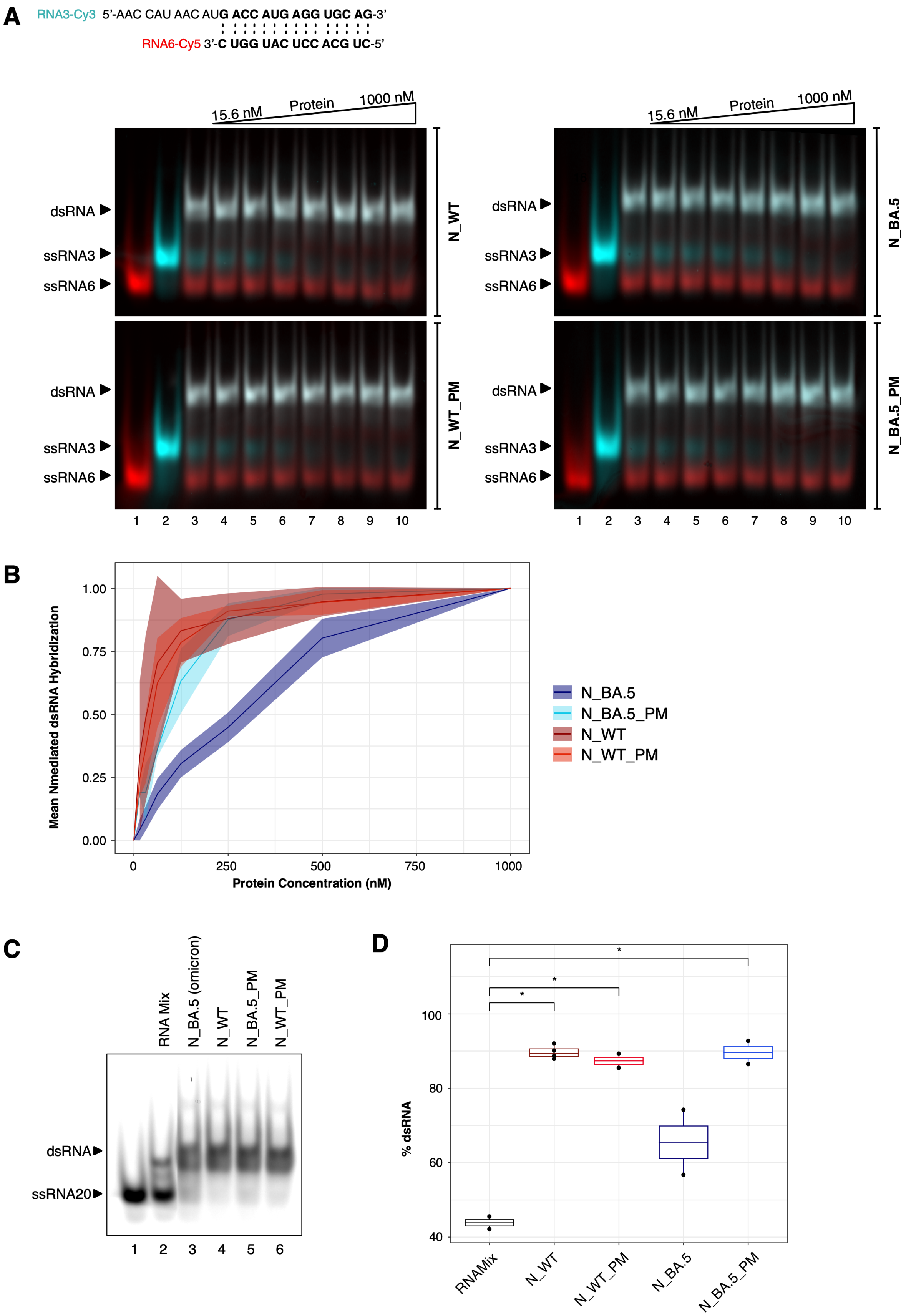

Supplementary Figure 3: Differences in RNA chaperone activity between Wuhan and Omicron BA.5 N and their phosphomimetic variants

(A) The RNA chaperone activity of N_WT, N_BA.5, and their phosphomimetic variants, N_WT_PM and N_BA.5_PM, was compared using an RNA Mix consisting of ssRNA3-Cy3 and ssRNA6-Cy5. The RNA mix was incubated with increasing protein concentrations (15–1000 nM, lanes 4-10). N_WT and N_WT_PM achieved complete RNA hybridization at 125 nM (lane 7). In contrast, N_BA.5 required 500 nM for complete hybridization (lane 9), whereas N_BA.5_PM restored RNA annealing efficiency, achieving complete hybridization at 125 nM (lane 7). Number of replicates n=3. **(B)** Quantification of chaperone assys shown in (A). dsRNA band intensities from two biological replicates of the chaperone assays, exhibiting identical experimental conditions, were quantified using Fiji. Normalized dsRNA hybridization values (y-axis) were plotted against protein concentration (x-axis) for N_WT, N_BA.5, N_WT_PM, and N_BA.5_PM. Each series was normalized to the RNA mix control, with normalized values shown as a thick line and standard deviation as a ribbon. N_BA.5 (dark blue) exhibited the weakest dsRNA hybridization efficiency. **(C)** The RNA chaperone activity of Wuhan and Omicron BA.5 N proteins, along with their phosphomimetic variants in the context of RNA20/RNA21, was compared at equimolar concentrations using an RNA Mix (ssRNA20-Cy5, ssRNA21). The mix was incubated for 3 minutes at 25°C in the absence (lane 2) or presence of 250 nM N_BA.5 (lane 3), N_WT (lane 4), N_BA.5_PM (lane 5), or N_WT_PM (lane 6). The samples were then analyzed on a 6% native PAA gel and visualized using the Typhoon^{TM} FLA 9500 reader. The RNA Mix alone (lane 2) served as a control for spontaneous RNA hybridization, while N_WT (lane 4) represented the fully hybridized state. N_WT_PM (lane 6) and N_BA.5_PM (lane 5) also demonstrated near-complete RNA annealing, whereas N_BA.5 (lane 3) showed only partial hybridization. Number of replicates n=3. **(D)** Boxplot representing the percentage of hybridized dsRNA shown in (C). Each boxplot represents the interquartile range (IQR), with the median indicated by a horizontal line inside the box. The upper and lower whiskers extend to a maximum of 1.5 times above and below the upper and lower quartiles, respectively. The x-axis represents the respective N truncation protein, while the percentage of dsRNA is indicated on the y-axis. Except for N_BA.5, each protein displayed a significant enhancement in RNA hybridization relative to the RNA mix control. The difference between N_BA.5 and the other proteins appears not significant. Non-significant comparisons are excluded in the illustration. Statistical analysis was performed by applying a t-test using the Benjamini-Hochberg (BH) procedure. '*' = 0.05 <= p >0.01.
